# Evaluating Wearable Electrodermal Activity as a Candidate Digital Biomarker of Occupational Well-Being: Longitudinal Real-World Study

**DOI:** 10.2196/104167

**Published:** 2026-11-11

**Authors:** Henna Salonius, Pasi Kytölä, Satu Pakarinen, Anne Mäkikangas, Virpi Liukkonen, Saija Mauno

**Affiliations:** 1 Faculty of Social Sciences, Tampere University, Kalevantie 4, Tampere, Pirkanmaa, 33100, Finland, +358 294 5211; 2 Work Research Centre, Tampere University, Tampere, Pirkanmaa, Finland; 3 Faculty of Information Technology and Communication Sciences, Tampere University, Tampere, Pirkanmaa, Finland; 4 Finnish Institute of Occupational Health, Helsinki, Uusimaa, Finland; 5 Faculty of Medicine and Health Technology, Tampere University, Tampere, Pirkanmaa, Finland; 6 Department of Psychology, University of Jyväskylä, Jyväskylä, Central Finland, Finland

**Keywords:** electrodermal activity, wearable sensors, ecological momentary assessment, digital biomarkers, occupational well-being, burnout, engagement, autonomic nervous system, monitoring, longitudinal study

## Abstract

**Background:**

Wearable physiological sensing and mobile self-report methods offer new opportunities for monitoring occupational well-being in real-world settings. However, interpreting continuous physiological signals in relation to psychological states such as job burnout and work engagement remains challenging, particularly when using deployable systems that rely on proprietary signal processing.

**Objective:**

This study examined a proprietary electrodermal activity (EDA)–derived arousal index as a candidate digital biomarker for research on occupational well-being. We examined associations between job burnout, work engagement, and sympathetic activation indexed by a device-derived EDA metric, and assessed whether combining physiological data with mobile-based ecological momentary assessment (mEMA) of valence provides complementary information.

**Methods:**

A total of 75 knowledge workers participated, with sufficient paired physiological and mEMA data obtained from 57 participants in wave 1 and 51 in wave 2 (total observations=108). Participants were monitored continuously over 14 consecutive days at 2 time points 6 months apart. Autonomic arousal was operationalized using the device-derived double normalized electrodermal activity (DNE) index obtained from wearable EDA recordings. Valence was assessed using mEMA. Associations between physiological activation, valence, and occupational well-being outcomes were analyzed using generalized least squares models with a compound-symmetry covariance structure to account for repeated observations within participants.

**Results:**

The wearable-derived DNE index showed a clear diurnal pattern, with higher values during daytime and lower values during nighttime. Greater within-nighttime DNE variability was associated with higher burnout (estimate=0.02, 95% CI 0.01-0.03; *P*<.001) and lower engagement (estimate=−0.03, 95% CI −0.05 to −0.01; *P*=.009). The daily DNE × valence interaction was also associated with engagement (estimate=−0.35, 95% CI −0.69 to −0.00; *P*=.047). Removing all DNE and valence variables significantly worsened model fit for both burnout (*χ*²_₁₀_=28.5; *P*=.002) and engagement (*χ*²_₁₀_=23.7; *P*=.009), supporting their combined explanatory value. Model-based profiles further showed that negative valence was associated with higher predicted burnout and lower predicted engagement than positive valence across both high- and low-arousal scenarios, although prediction intervals were wide and partially overlapping. Model comparisons showed that including physiological and mEMA-based variables improved explanatory power.

**Conclusions:**

These findings provide practical evidence that continuous wearable EDA monitoring can be combined with mEMA in naturalistic occupational settings. Associations between the proprietary DNE index, valence, burnout, and work engagement provide preliminary convergent evidence supporting further evaluation of DNE as a candidate digital biomarker in occupational well-being research. Interpretation remains constrained by the proprietary signal-processing pipeline, and this study does not establish construct-specific validity, prospective predictive performance, screening or diagnostic validity, sensitivity to change, or individual-level monitoring usage.

## Introduction

Research on occupational well-being has traditionally relied on retrospective self-report questionnaires and single-time-point assessments, which may not adequately capture the dynamic and context-dependent nature of burnout and engagement. Such approaches are subject to recall bias and limited ecological validity. Physiological approaches have provided additional insight, but these have often been restricted to laboratory settings or short-term measurements, limiting their applicability to everyday working life. Recent advances in wearable and mobile technologies provide new opportunities to address these challenges. Digital health approaches combining wearable sensing with mobile-based self-report enable continuous monitoring of physiological and psychological processes in real-world settings [1,2]. Compared with traditional approaches relying primarily on retrospective questionnaires and single-time-point assessments, such methods may provide more timely and ecologically valid insights into occupational well-being and burnout-related processes [3-5].

Job burnout (hereafter burnout), a prolonged response to chronic occupational stress, is commonly defined as a work-related syndrome characterized by exhaustion, cynicism toward work, and reduced professional efficacy [6-8]. Burnout has been associated with a range of adverse outcomes, including cardiovascular disease, depression, and absenteeism [9], highlighting its relevance for both organizational and public health. Work engagement (hereafter engagement), in contrast, is typically defined as a positive, work-related state characterized by vigor, dedication, and absorption [7,10,11]. Engagement has been linked to beneficial outcomes, and fostering engagement is therefore considered desirable [12]. Although burnout and engagement are often conceptualized as opposite ends of a well-being continuum [11], their relationship remains debated. Some evidence suggests that they are distinct constructs, whereas other research indicates that they are highly negatively correlated and may share common antecedents [13]. Moreover, individuals may simultaneously experience elements of both states, such as exhaustion and vigor at the average level [14,15].

This ongoing debate extends to their psychophysiological correlates, where findings have been inconsistent and, in some cases, contradictory [5,16-18]. Recent reviews have highlighted that biomarker findings in burnout research are highly sensitive to sampling timing, circadian factors, and methodological heterogeneity, underscoring the need for ecologically valid longitudinal assessment approaches capable of capturing physiological dynamics in everyday settings [19]. Despite extensive research, the physiological underpinnings of burnout and engagement remain insufficiently understood.

To address the aforementioned gaps, this study examines electrodermal activity (EDA) as a wearable physiological signal indexing sympathetic autonomic activation in knowledge workers monitored over 14 consecutive days. Because EDA is driven by eccrine sweat gland activity under sympathetic control, it is commonly used as an indicator of autonomic arousal [20]. To support interpretation of physiological data, we draw on the circumplex model of occupational well-being [21-23], which conceptualizes affective states along 2 dimensions: autonomic arousal (activation-deactivation) and valence (pleasant-unpleasant). In this study, this framework is adapted to focus on occupational mental health outcomes. Specifically, high arousal combined with negative valence is interpreted as reflecting a state of increased risk of burnout, whereas low arousal combined with negative valence may represent more severe states of exhaustion or depressive symptomatology. In contrast, high arousal and positive valence correspond to engagement, while low arousal and positive valence reflect a state of work-related contentment. This 2-dimensional framework originally introduced by Russell [24] provides a conceptual basis for integrating physiological activation and subjective experience in the interpretation of occupational well-being in real-world settings. Within this framework, the arousal dimension was operationalized using wearable EDA measurements, while valence was assessed using mobile-based ecological momentary assessment (mEMA), a method that captures individuals’ experiences in real time or near real time within their natural environments, thereby reducing recall bias and increasing ecological validity [25]. The combined approach enables examination of how physiological activation and subjective experience jointly relate to occupational well-being over time. The adapted framework is illustrated in Figure 1.

**Figure 1.**
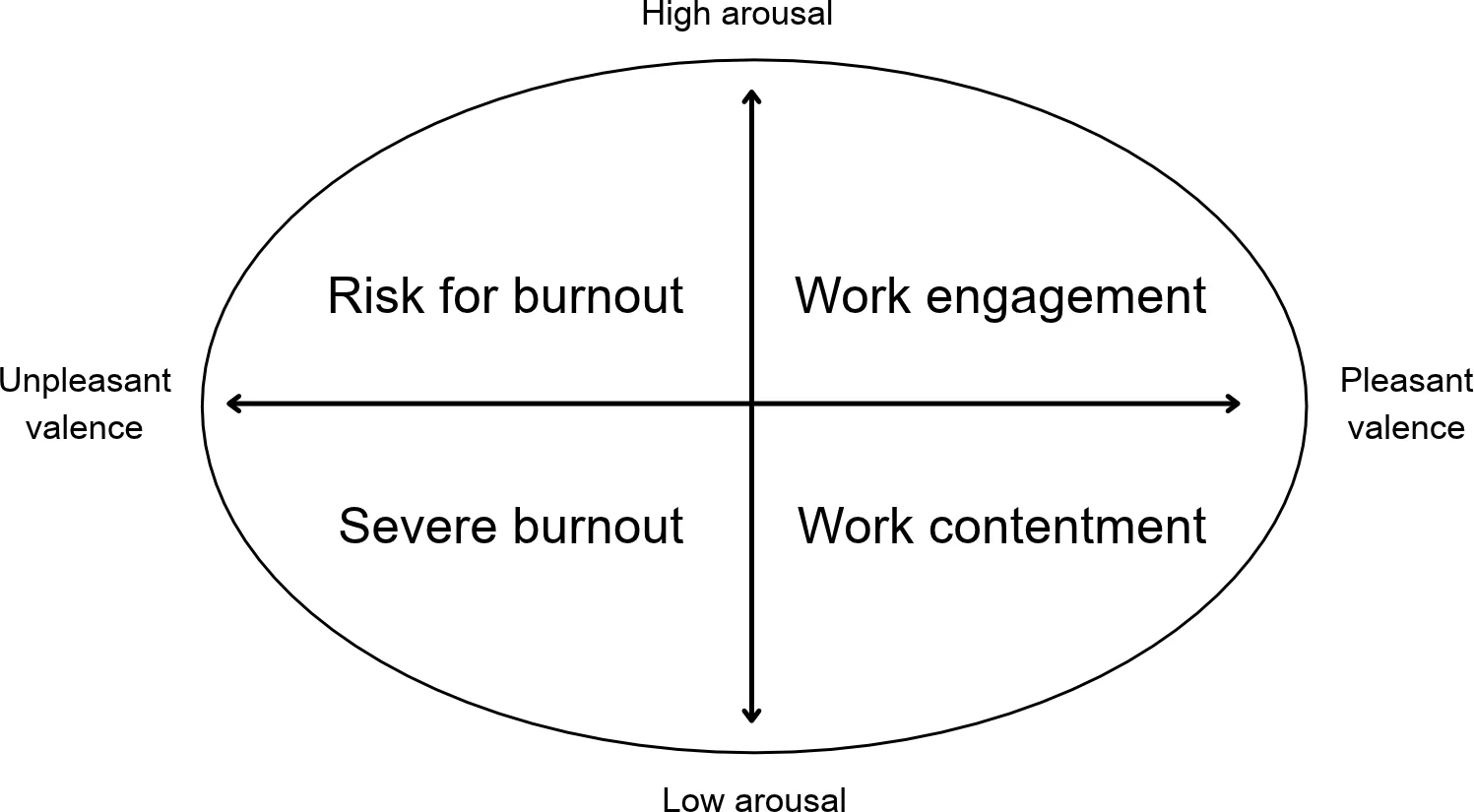
Adapted circumplex model of occupational well-being [21-23]. The figure illustrates occupational well-being along dimensions of autonomic arousal and valence. In this study, arousal was indexed using wearable electrodermal activity, and valence was assessed using mobile-based ecological momentary assessment. The model distinguishes between engagement, work contentment, risk of burnout, and more severe burnout.

Although wearable EDA approaches have individually been applied in stress and affective research [26], few studies appear to have integrated continuous long-term EDA monitoring with ecological momentary assessment (EMA)–based assessment of valence in naturalistic occupational settings. Recent longitudinal studies have demonstrated the feasibility of combining wearable sensor data and EMA to monitor burnout-related processes during return-to-work trajectories [3]. However, these approaches have primarily focused on sleep, activity, mobility, and smartphone-derived behavioral indicators rather than continuous electrodermal measures of autonomic arousal. Despite increasing interest in wearable and EMA approaches in occupational health research, relatively few studies have specifically integrated continuous long-term EDA monitoring with EMA-based assessment of valence in naturalistic occupational settings.

The aim of this study was to explore the use of continuous wearable EDA in occupational well-being research and to examine whether temporal patterns of the device-derived arousal index were associated with burnout and engagement. In addition, we explored whether combining EDA with mEMA-based assessments of valence provides complementary information consistent with the circumplex framework. Burnout and engagement were treated as continuous constructs in the primary analyses, while the circumplex model served as a conceptual guide. To our knowledge, this is among the first studies to examine continuous long-term EDA as a wearable-derived indicator of autonomic arousal in relation to burnout and engagement in a naturalistic occupational setting, while integrating EMA-based valence assessment within a circumplex framework of occupational well-being. This study extends previous work by evaluating a deployable wearable-derived metric in a real-world occupational setting, rather than relying exclusively on laboratory-based or acute stress–based physiological assessment [18,27]. In addition to examining associations between autonomic activation and occupational well-being, this study contributes to broader methodological discussions regarding the evaluation of wearable-derived digital measures. Recent work has highlighted both the challenges associated with proprietary wearable systems and the need for rigorous validation frameworks when direct reference standards or access to underlying algorithms are limited [28,29]. In this context, the term candidate digital biomarker denotes a digitally derived physiological measure under exploratory evaluation; it does not imply established construct-specific, predictive, diagnostic, or clinical validity. By evaluating a wearable-derived autonomic activation metric against established burnout and engagement measures, this study provides preliminary convergent evidence regarding associations between a wearable-derived autonomic arousal metric and established measures of occupational well-being.

## Methods

### Study Design

This study used a longitudinal follow-up design with repeated measurements collected at 6-month intervals between March 2022 and September 2022. The full study protocol included 4 waves; however, at the time of analysis, data from the first 2 waves were available and included in this study. Each measurement wave consisted of a 14-day ambulatory monitoring period, during which participants provided continuous physiological data and mEMA, followed by a postmeasurement questionnaire. The study was conducted in naturalistic work environments to examine real-world patterns of occupational well-being. The primary objective was to investigate associations between EDA, valence, burnout, and engagement. The analytic sample included participants with sufficient paired physiological and self-report data across the first 2 waves (wave 1: n=57; wave 2: n=51; total observations=108). The study design reflects a digital health-monitoring approach combining wearable sensing and mobile-based self-report in real-world settings.

### Participants and Recruitment

Participants were recruited from 3 Finnish knowledge-intensive companies. A total of 123 employees enrolled in the study, of whom the majority were Finnish-speaking (n=119, 96%), while a small proportion were of other nationalities (n=4, 3%) with English as their working language. Participants were invited via email and provided with detailed study information in Finnish and English. Eligibility criteria included being employed by one of the participating organizations at the start of the study and having no significant self-reported health conditions that would prevent participation. Individuals with known nickel allergy were advised not to participate due to potential skin reactions to the wearable device. Symptoms of anxiety and depression were not exclusion criteria but were assessed and included as covariates in the analyses.

Participation was voluntary, and all participants provided informed consent prior to data collection. No financial compensation was provided. The average duration of work experience in the current position was 7.1 (SD 6.8) years in wave 1 and 6.1 (SD 5.7) years in wave 2. Detailed demographic, occupational, behavioral, and psychological characteristics of the analytic sample are reported separately for each measurement wave in Table S2 in Multimedia Appendix 1, including age, sex, working hours, family commitments, sleep duration, medication affecting sympathetic nervous system activity, managerial position, work experience, nicotine use, BMI, anxiety symptoms, and depressive symptoms.

### Data Collection Procedure

Data collection was conducted in naturalistic work settings and followed a systematic sequence within each measurement wave. Prior to the first measurement wave, participants were provided with a wearable device and access to a mobile app that was used to collect both physiological data from the device and mEMA responses. Participants also received instructions for using both the device and the app. For subsequent waves, participants retained the devices and were informed by email about the upcoming data collection period and instructed to resume the same measurement protocol.

Each measurement wave consisted of a 14-day monitoring period. During this time, participants continuously wore the wearable device to record EDA during both daytime activities and nighttime sleep. Participants were instructed to wear the device for at least 16 hours per day to ensure sufficient data coverage. In parallel with physiological monitoring, participants completed mEMA of valence using a mobile app. Prompts were delivered every 3 hours during waking hours, and participants were asked to report their overall emotional experience during the preceding interval. Responses could be entered retrospectively within a 12-hour window, allowing participants to maintain normal sleep patterns without interruption. Following the 14-day monitoring period, participants were invited to complete an online questionnaire assessing burnout, engagement, and relevant background variables. The questionnaire was designed to capture experiences over the same 2-week period as the physiological and mEMA data collection. This timing was intentional, as the validated self-report measures used in the study assess experiences retrospectively over approximately 2-week periods, allowing alignment between the temporal scope of the questionnaire data and the physiological monitoring window. Data quality criteria were predefined to ensure reliable physiological measurement. Days with less than 16 hours of wearable data were excluded, and participants with fewer than 6 valid days of data within a measurement wave were excluded from analyses. This multimodal data collection approach enabled the integration of continuous physiological monitoring, repeated momentary self-reports, and retrospective questionnaire data within the same real-world observation period. Figure 2 illustrates the data collection workflow within each measurement wave.

**Figure 2.**
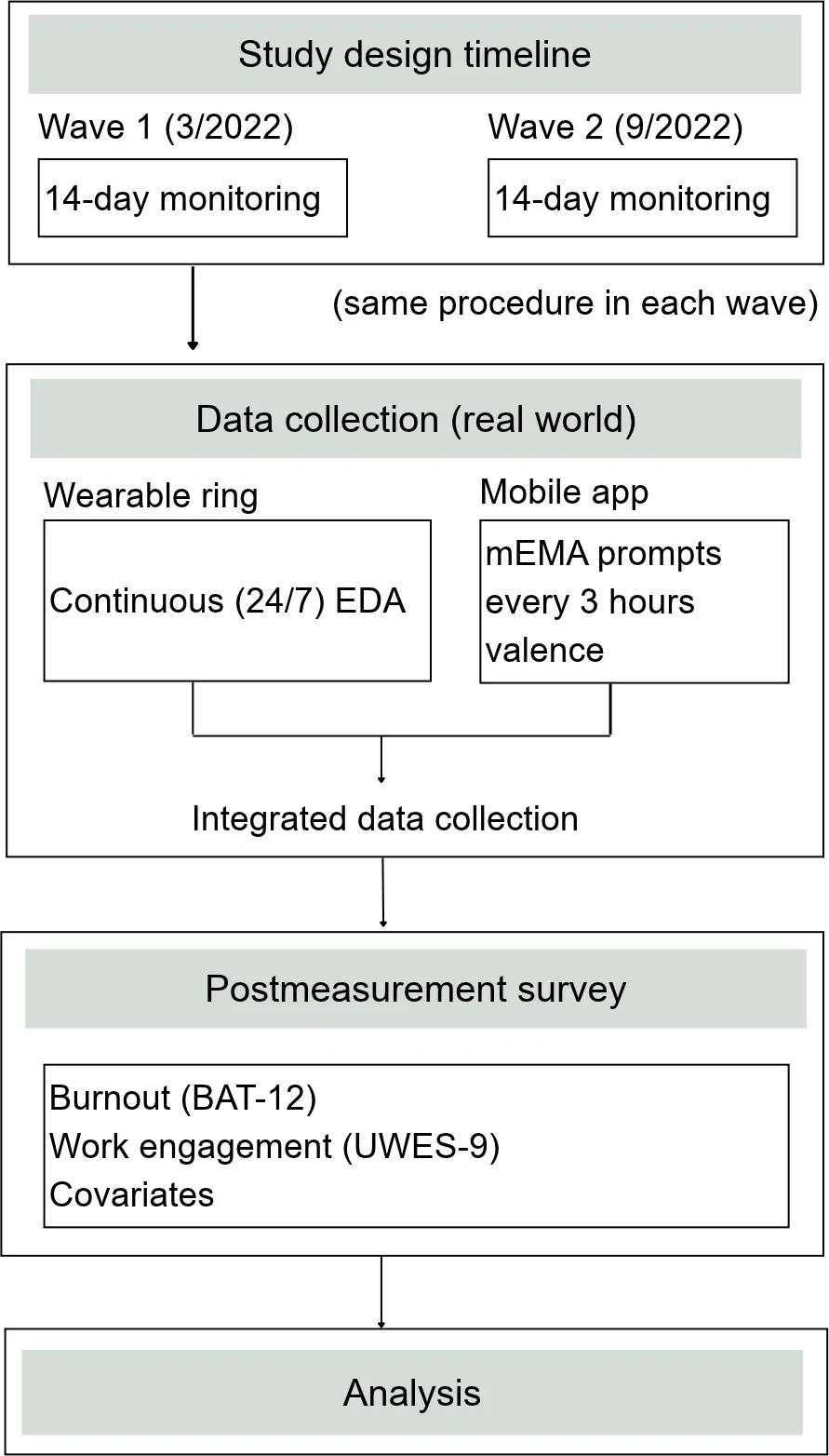
Data collection workflow across measurement waves. BAT-12: 12-item Burnout Assessment Tool; EDA: electrodermal activity; mEMA: mobile-based ecological momentary assessment; UWES-9: 9-item Utrecht Work Engagement Scale.

### Ethical Considerations

The study was conducted in accordance with the principles of the Declaration of Helsinki. Ethical approval was obtained from the Regional Ethics Committee of the Expert Responsibility Area of Tampere University Hospital (identifier R21149). Participation was voluntary, and all participants provided informed consent prior to enrollment. Participants received no financial or other compensation for their participation. Participants were informed about the purpose of the study, the types of data collected (including continuous physiological data and self-reported measures), and their right to withdraw from the study at any time without consequences.

Given the use of wearable devices and mobile apps in a workplace context, particular attention was paid to data privacy and participant autonomy. Physiological and self-report data were collected for research purposes only and were not shared with employers at the individual level. Data were deidentified prior to analysis, and all results are reported at an aggregated level. Data-handling procedures complied with applicable data protection regulations, including the General Data Protection Regulation.

### Measures

#### Wearable Physiological Data EDA

Continuous EDA data were collected using a commercially available ring-type wearable device (Nuanic Ring [version V7AC_2; formerly Moodmetric smart ring; Nuanic Oy]). Participants wore the ring on a finger of their choice during both daytime activities and sleep, with the electrodes contacting palmar skin. The device measures skin conductance between 2 silver-coated electrodes using an exosomatic alternating-current method (1.8 Vp-p; shunt resistor 1 MΩ). Previous independent studies have evaluated the signal measurement performance and field use of the ring, reporting approximately 83% agreement with laboratory-grade EDA measurement [30,31]. These findings concern the EDA measurement system and should not be interpreted as validation of the proprietary double normalized electrodermal activity (DNE) index.

The device is capable of recording raw EDA at 3‐16 Hz during short-term measurements. For the continuous 14-day monitoring used in this study, the manufacturer’s software provided the DNE index at 1-minute intervals. DNE is scaled from 1 to 100, with higher values representing higher device-estimated autonomic activation. The algorithm normalizes EDA relative to individual-specific typical values accumulated during device use, and a 12-hour initial usage period was completed before each measurement period as recommended by the manufacturer.

The DNE-processing algorithm is proprietary. It is designed to reduce the influence of measurement artifacts and sources of variation such as motion, skin contact, temperature, wear characteristics, and intra- and interindividual differences; however, the specific preprocessing, filtering, artifact detection, wear detection, and normalization procedures were not accessible to the investigators. These processing steps therefore cannot be independently evaluated or reproduced from the available data. Accordingly, DNE is treated in this study as a proprietary EDA-derived arousal index under exploratory evaluation rather than as an independently validated or construct-specific measure of burnout or engagement.

However, this study evaluates a wearable-derived physiological metric produced by a proprietary signal-processing pipeline rather than a fully transparent physiological signal-processing system. This distinction reflects a broader methodological consideration in digital health research, where many scalable and user-facing systems rely on proprietary signal-processing pipelines. In such contexts, where direct access to proprietary data-processing procedures may be limited, evaluation can instead characterize the measurement performance and behavior of wearable-derived metrics in real-world settings and examine their associations with established measures, while explicitly acknowledging the limits imposed by inaccessible algorithms [28,29].

Accordingly, this study focuses on characterizing the behavior of the available DNE output and its associations with established measures rather than attempting to reconstruct the proprietary signal-processing pipeline. Although the proprietary nature of the index limits transparency, emerging evidence provides preliminary evidence regarding its relationship with autonomic activation. For example, a recent study [32] reported that long-term DNE monitoring was associated with sympathicotonia and related physiological symptoms, suggesting that the index captures meaningful variations in sympathetic nervous system activity. While this does not constitute formal validation of the DNE index as a construct-specific measure, it provides supportive evidence for its use as a proxy of autonomic activation in ambulatory conditions. To support transparency and ease of reuse, deidentified derived data and R code will be made publicly available via the Finnish Social Science Data Archive Aila Data Service. The device and software version used in this study is not the current release, which may limit direct replication with newer versions. The device and software configuration used to generate the DNE index remained unchanged throughout the study. No software or device updates affecting DNE calculation were applied between the measurement waves. For this device version, such changes would have required the rings to be physically returned to Nuanic Oy for reprogramming, which did not occur during the study.

#### mEMA of Valence

Valence refers to the pleasantness or unpleasantness of an emotional experience and was assessed using a single-item near-real-time mEMA question delivered via the mobile app associated with the wearable device. Participants received prompts every 3 hours during waking hours and were asked to rate their overall emotional experience during the preceding interval (eg, “How did you feel today between 9 AM and 12 PM?”). Responses were recorded on a 7-point scale ranging from 1 (“very unpleasant”) to 7 (“very pleasant”), with 4 representing a neutral response. Participants were allowed to respond retrospectively within a 12-hour window, enabling uninterrupted sleep during nighttime periods.

For the main regression analyses and construction of the illustrative model-based profiles, individual mEMA reports were not temporally matched to concurrent DNE observations. Instead, valence was summarized for each participant and measurement wave as the median of the available mEMA responses across the 14-day monitoring period. The DNE predictors were likewise derived as participant-wave-level summaries of the physiological data collected during the same monitoring period. The resulting valence measure was treated as a numerical explanatory variable in the statistical analyses.

#### Self-Report Measures of Burnout, Engagement, and Covariates

Following each 14-day monitoring period, participants completed a questionnaire assessing burnout, engagement, and relevant covariates. Burnout was measured using the 12-item Burnout Assessment Tool (BAT-12), which assesses 4 dimensions: exhaustion, cognitive impairment, emotional impairment, and mental distancing [33,34]. Responses are given on a 5-point scale (1=never to 5=always), and a composite score was used in the analyses. Engagement was assessed using the 9-item Utrecht Work Engagement Scale (UWES-9), which measures vigor, dedication, and absorption [35]. Responses are given on a 7-point scale (0=never to 6=always or every day), and a composite score was used.

Symptoms of anxiety and depression were assessed using the Generalized Anxiety Disorder 7-item scale (GAD-7) [36] and the Beck Depression Inventory-II (BDI-II) [37], respectively, as part of the postmeasurement questionnaire. Their scoring categories and cutoff values are reported in Table S1 in Multimedia Appendix 1. Additional demographic, occupational, behavioral, and health-related covariates are described in Table S2 in Multimedia Appendix 1. Physical activity was estimated from wearable-derived activity data normalized for wear time. These variables were included as covariates in the statistical models. Internal consistency of the BAT-12 and UWES-9 scales was high across both measurement waves (Cronbach α range 0.87‐0.96).

### Data Processing and Feature Extraction

Continuous EDA data were processed using the device manufacturer’s software to derive minute-level DNE time series. A 12-hour initial usage period was completed prior to each measurement period, as recommended by the manufacturer. Data quality control followed predefined wear-time criteria: days with less than 16 hours of valid data were excluded, and participants with fewer than 6 valid days per measurement period were excluded from the analyses.

From the DNE time series, summary variables were derived to characterize patterns of autonomic activation. These included indicators of daily average activation, between-day variability, and activation levels during waking and rest periods, as well as within-period variability (Table 1). For each valid 24-hour period (6 AM to 6 AM), daily DNE was calculated as the mean of the minute-level DNE values, and these daily values were averaged across the measurement period. Between-day variation was defined as the standard deviation of these daily means. Nighttime (11 PM to 7 AM) and daytime (7 AM to 11 PM) DNE levels were calculated analogously. Within-nighttime and within-daytime variations were defined as the average across days of the interquartile range of minute-level DNE values within the corresponding time interval. These derived variables were used as predictors in the statistical models. As a physiological plausibility check, diurnal patterns of the DNE index were examined, including expected reductions during nighttime periods. These patterns are reported in the Results section.

**Table 1.** Main regression analysis results for burnout and engagement^a^.

Variable	Burnout estimate (95% CI)	*P* value	Engagement estimate (95% CI)	*P* value
Intercept	7.05 (0.90 to 13.20)	.03	−4.02 (−17.19 to 9.15)	.55
Daily DNE^b^ level	−0.77 (−1.52 to −0.02)	.045	1.82 (0.18 to 3.45)	.03
Daily DNE-level variation	0.03 (−0.01 to 0.06)	.10	−0.05 (−0.11 to 0.02)	.17
Nighttime DNE level	0.22 (−0.07 to 0.50)	.13	−0.57 (−1.19 to 0.05)	.07
Within nighttime DNE-level variation	0.02 (0.01 to 0.03)	<.001	−0.03 (−0.05 to −0.01)	.009
Daytime DNE level	0.44 (−0.06 to 0.95)	.08	−1.09 (−2.19 to 0.01)	.052
Within daytime DNE-level variation	−0.01 (−0.02 to 0.00)	.15	0.01 (−0.03 to 0.04)	.69
Valence	−1.19 (−2.49 to 0.12)	.07	1.83 (−0.96 to 4.62)	.20
Physical activity	−0.02 (−0.11 to 0.07)	.68	0.16 (−0.04 to 0.36)	.12
Age (years)	−0.01 (−0.03 to 0.01)	.32	0.001 (−0.04 to 0.04)	.97
Sex (male)	−0.13 (−0.34 to 0.08)	.22	−0.21 (−0.68 to 0.26)	.38
Working hours per week	0.004 (−0.02 to 0.03)	.67	0.01 (−0.04 to 0.05)	.81
Commitments, underage, Yes	−0.12 (−0.33 to 0.10)	.28	−0.10 (−0.58 to 0.38)	.68
Commitments, adults, Yes	−0.30 (−0.78 to 0.19)	.23	0.98 (−0.06 to 2.02)	.07
Major life event, Yes	−0.09 (−0.35 to 0.17)	.49	0.36 (−0.19 to 0.92)	.19
Need change, Yes	−0.04 (−0.28 to 0.20)	.73	−0.13 (−0.65 to 0.38)	.61
Intervention, Yes	0.27 (−0.06 to 0.61)	.11	−0.53 (−1.28 to 0.22)	.16
Sleep time work	−0.10 (−0.23 to 0.03)	.12	0.21 (−0.07 to 0.48)	.14
Sleep time leisure	0.04 (−0.09 to 0.16)	.56	−0.06 (−0.33 to 0.22)	.69
Medication, SNS^c^, Activates	0.16 (−0.21 to 0.53)	.40	0.12 (−0.69 to 0.93)	.77
Managerial position, Yes	0.23 (−0.03 to 0.49)	.08	0.13 (−0.44 to 0.70)	.65
Experience (years)	0.01 (−0.01 to 0.02)	.40	−0.01 (−0.05 to 0.02)	.45
Nicotine, Yes	−0.31 (−0.87 to 0.25)	.27	0.78 (−0.48 to 2.04)	.22
BMI	−0.001 (−0.02 to 0.02)	.96	0.06 (0.02 to 0.11)	.01
Anxiety (GAD-7^d^ total score)	0.02 (−0.01 to 0.05)	.22	−0.04 (−0.11 to 0.02)	.17
Depression (BDI-II^e^ total score)	0.05 (0.03 to 0.07)	<.001	−0.08 (−0.12 to −0.04)	<.001
Interaction, daily DNE × valence	0.15 (−0.00 to 0.31)	.052	−0.35 (−0.69 to −0.00)	.047
Interaction, night DNE × valence	−0.04 (−0.10 to 0.02)	.15	0.11 (−0.02 to 0.24)	.11
Interaction, day DNE × valence	−0.09 (−0.19 to 0.02)	.10	0.20 (−0.03 to 0.43)	.08

a Burnout model correlation structure: compound symmetry, *ρ*=0.66. Residual standard error σ=0.41. Degrees of freedom: 108 total; 79 residual. Engagement model correlation structure: compound symmetry, *ρ*=0.72. Residual standard error σ=0.92. Degrees of freedom: 108 total; 79 residual. All coefficients are unstandardized.

b DNE: double normalized electrodermal.

c SNS: sympathetic nervous system.

d GAD-7: Generalized Anxiety Disorder 7-item scale.

e BDI-II: Beck Depression Inventory-II.

### Statistical Analysis

#### Descriptive Analyses

Descriptive analyses were conducted to characterize the data and examine preliminary relationships between variables. Pearson correlation coefficients were calculated to assess the association between burnout and engagement at each measurement wave. To visualize patterns of autonomic activation, average hourly levels of the DNE index were calculated and plotted across the 24-hour cycle for each measurement wave.

#### Main Regression Analyses

To examine associations between EDA, valence, and occupational well-being outcomes, linear models for correlated data were fitted using generalized least squares (GLS) with restricted maximum likelihood (REML) estimation. Main regression analyses were based on complete participant-wave records containing valid EDA, mEMA valence, and questionnaire data. No missing values were imputed. Detailed data availability by study component and measurement wave is reported in Table S3 in Multimedia Appendix 1. Because 33 participants contributed observations at both measurement waves, participant-wave observations were not assumed to be independent. We therefore fitted GLS models for correlated data using REML estimation. A compound-symmetry covariance structure allowed a common nonzero correlation between repeated observations from the same participant while assuming independence between participants. Data from both waves were analyzed jointly because the objective was to examine associations across measurement occasions rather than temporal change; wave was therefore not included as a fixed effect, and wave-by-predictor interactions were not evaluated. The regression models were adjusted for a broad set of demographic, behavioral, sleep-related, and psychological covariates to evaluate whether DNE-related variables were associated with burnout beyond established and plausible confounding factors. The purpose of the analysis was not to derive a parsimonious predictive model but to assess whether DNE-related variables retained explanatory value after multivariable adjustment. The models were associational and were not intended to estimate causal effects.

Burnout and engagement were treated as continuous outcome variables derived from validated questionnaire scores. The main explanatory variables included DNE-related variables, valence, and relevant covariates (eg, demographic and health-related variables). Continuous predictors were entered in their original measurement units and were neither standardized nor centered. DNE variables were therefore analyzed on their original scale, and valence was treated as a numerical predictor on its original 1‐7 scale. Categorical covariates were indicator coded, and the reported regression coefficients are unstandardized. The *P* values of the REML estimates for the model fixed parameters were based on *t*-approximation. Residual plots were inspected for approximate normality and homoscedasticity, with no major departures observed.

To evaluate whether DNE- and valence-related variables contributed explanatory value beyond conventional covariates, full models including these variables were compared with reduced models excluding selected DNE and/or valence variables using likelihood ratio test type test statistic. Model comparisons were conducted using maximum likelihood estimation. Inferential conclusions were based on 2-sided tests at the 95% CI.

#### Illustrative Model-Based Profiles

To aid interpretation of the regression models, illustrative model-based profiles were generated for hypothetical covariate profiles representing combinations of high versus low arousal and negative versus positive valence, while holding other covariates at their observed mean values. The DNE values representing high- and low-arousal profiles were selected from the lower and upper regions of the observed distributions after inspection of the 5th, 10th, 15th, 85th, 90th, and 95th percentiles. The exact predictor values used for the profiles are reported in Table S6 in Multimedia Appendix 1. These profiles were intended as illustrative scenarios for visualizing the joint relationships between arousal, valence, and occupational well-being outcomes, rather than as realistic predictions for specific individuals or as an empirical classification of participant states. The predictor used was the GLS predictor based on REML-estimated covariance parameters. The approximately normal residuals supported the normality assumption underlying the REML estimation. Predicted outcome values were accompanied by asymptotic 80% prediction intervals, illustrating the expected range of values under the specified conditions. The 80% prediction intervals were used descriptively to illustrate predictive uncertainty and overlap between the hypothetical profiles; they were not used for fixed-effect hypothesis testing for which 2-sided inference at the 95% CL was used.

#### Time-Resolved Analyses

To examine temporal patterns in autonomic activation, hourly DNE profiles across the 24-hour cycle were analyzed using linear mixed-effects models. Diurnal variation was represented using the first 3 harmonics of a 24-hour cycle. This provided a parsimonious smooth representation of the daily DNE profile: the 24-hour component captured the broad day-night pattern, while the 12- and 8-hour harmonics allowed additional flexibility in profile shape without estimating a separate effect for each hour. Group-specific harmonic terms allowed the shape of the daily DNE profile to differ between burnout or engagement subgroups. Subject-specific random effects were included to account for repeated hourly measurements within individuals. The models were used to assess differences in hourly activation patterns across subgroups defined using established cutoff values.

Estimated marginal means were obtained for each hour of the day while holding depression and anxiety covariates at minimal levels. Pairwise comparisons between groups were conducted at each hour using 2-tailed t tests of estimated marginal mean contrasts, with *P* values adjusted using the Tukey method to control the family-wise error rate. Pointwise 95% CIs were calculated for all estimated marginal means. Given the moderate sample size, the small number of participants in some subgroups, and the number of model parameters, these analyses were interpreted as exploratory. All analyses were conducted in R (R Foundation for Statistical Computing) [38] using the *nlme* [39] and *emmeans* [40] packages.

## Results

### Sample Characteristics and Descriptive Findings

Adequate paired wearable, EMA, and questionnaire data were available for 57 participant-wave observations in wave 1 and 51 participant-wave observations in wave 2, resulting in 108 analyzable observations from 75 distinct participants (Figure 3). Complete datasets required valid wearable data, EMA responses, and completed surveys within the same measurement wave. Detailed data availability for each data source is presented in Table S3 in Multimedia Appendix 1. The analytic sample had a mean age of 39.2 (SD 7.0) years in wave 1 and 39.7 (SD 7.3) years in wave 2; the corresponding female/male distributions were 32/25 and 26/25. Mean GAD-7 scores were 5.4 (SD 4.1) and 5.8 (SD 3.7), and mean BDI-II scores were 8.7 (SD 6.7) and 9.4 (SD 7.5), respectively. Additional demographic, occupational, behavioral, and psychological characteristics are reported in Table S2 in Multimedia Appendix 1.

The distribution of burnout (BAT-12) and engagement (UWES-9) scores across the analytic sample (n=108 observations) is presented in Table 2. Burnout and engagement were strongly negatively correlated at both time points (wave 1: *r*=−0.69, 95% CI −0.81 to −0.52, *t*_55_=−7.08, *P*<.001; wave 2: *r*=−0.79, 95% CI −0.87 to −0.65, *t*_49_=−8.94, *P*<.001).

**Figure 3.**
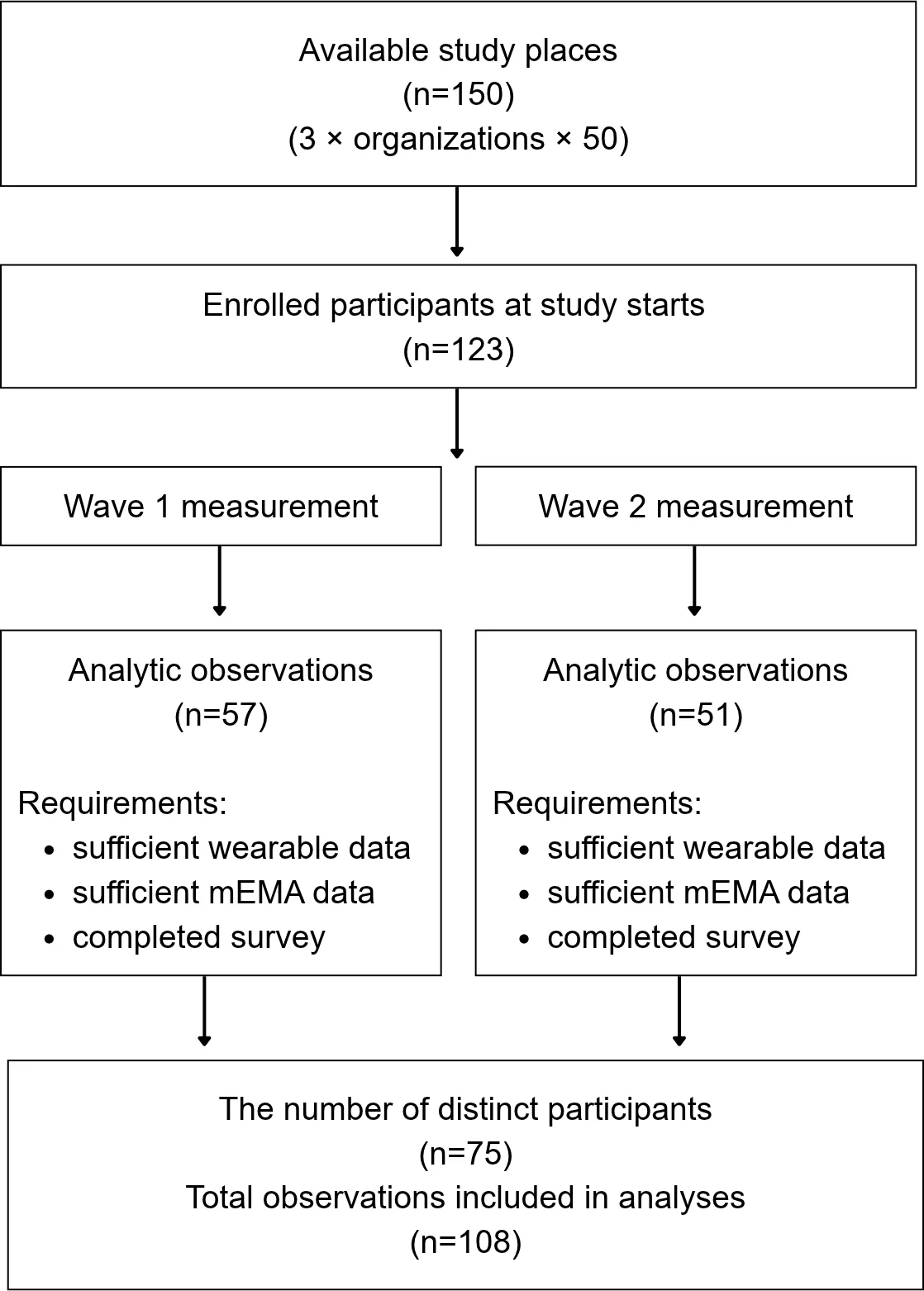
Participant flow diagram. The number of participants available at the start of each wave could not be reconstructed precisely from the available records. Analytic observations refer to participant-wave observations with sufficient wearable, EMA, and survey data. mEMA: mobile-based ecological momentary assessment.

To examine overall patterns of autonomic activation, average hourly levels of the DNE index were analyzed across the 24-hour cycle (Figures 4A and 4B). DNE levels increased in the morning (around 7 AM), remained elevated during daytime hours, and decreased in the late evening (around 11 PM), with an overall mean level of approximately 50.

**Table 2.** Burnout and engagement scores across the sample according to their cutoff values.

Scores	Wave 1, n (%)	Wave 2, n (%)
Burnout (BAT-12^a^)		
No risk of burnout (total score <2.54)	47 (82.5)	37 (72.5)
At risk for burnout (2.54 ≤ total score <3.04)	7 (12.3)	10 (19.6)
Burnout likely (total score ≥3.04)	3 (5.2)	4 (7.9)
Total	57 (100)	51 (100)
Engagement (UWES-9^b^)		
Very low (total score ≤1.77)	1 (1.8)	3 (5.9)
Low (1.77 < total score ≤2.88)	3 (5.3)	5 (9.8)
Moderate (2.88 < total score ≤4.66)	19 (33.3)	22 (43.1)
High (4.66 < total score ≤5.5)	25 (43.9)	15 (29.4)
Very high (total score >5.5)	9 (15.7)	6 (11.8)
Total	57 (100)	51 (100)

a BAT-12: 12-item Burnout Assessment Tool.

b UWES-9: 9-item Utrecht Work Engagement Scale.

**Figure 4.**
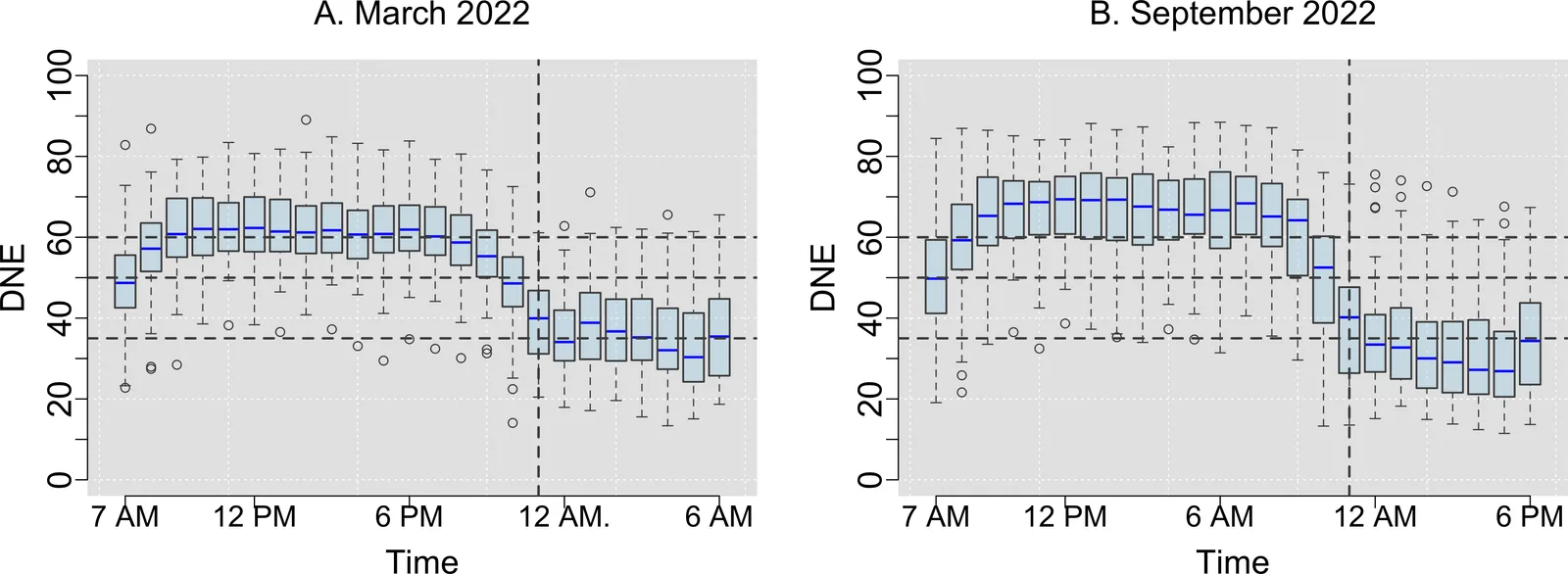
Participant’s hourly DNE-level variation over a day. (A) March 2022; (B) September 2022. DNE: double normalized electrodermal activity.

### Main Regression Analyses

Regression results for burnout and engagement are summarized in Table 1. Daily DNE was negatively associated with burnout (estimate=−0.77, 95% CI −1.52 to −0.02; *P*=.045). Valence showed a negative association with burnout that did not reach statistical significance (estimate=−1.19, 95% CI −2.49 to 0.12; *P*=.07). The interaction between daily DNE and valence was not statistically significant (estimate=0.15, 95% CI −0.00 to 0.31; *P*=.052), although the positive interaction coefficient suggested that the negative association between daily DNE and burnout was more pronounced at lower levels of valence and weaker at higher levels of valence. Greater within-nighttime variability in DNE was independently associated with higher burnout scores (estimate=0.02, 95% CI 0.01-0.03; *P*<.001). Higher daytime DNE levels showed a positive association with burnout that did not reach statistical significance (estimate=0.44, 95% CI −0.06 to 0.95; *P*=.08). In addition, higher depressive symptom scores (BDI-II) were strongly associated with higher burnout scores (estimate=0.05, 95% CI 0.03-0.07; *P*<.001).

In the engagement model, higher daily DNE levels were associated with higher engagement scores (estimate=1.82, 95% CI 0.18-3.45; *P*=.03). Nighttime DNE levels showed a negative association with engagement that did not reach statistical significance (estimate=−0.57, 95% CI −1.19 to 0.05; *P*=.07), and daytime DNE levels also showed a negative association with engagement that did not reach statistical significance (estimate=−1.09, 95% CI −2.19 to 0.01; *P*=.052). Greater within-nighttime variability in DNE was associated with lower engagement scores (estimate=−0.03, 95% CI −0.05 to −0.01; *P*=.009). The interaction between daily DNE and valence was statistically significant (estimate=−0.35, 95% CI −0.69 to −0.00; *P*=.047), indicating that the positive association between daily DNE and engagement was stronger at lower levels of valence and weaker at higher levels of valence. In addition, higher depressive symptom scores (BDI-II) were strongly associated with lower engagement scores (estimate=−0.08, 95% CI −0.12 to −0.04; *P*<.001).

Model comparison analyses in Table 3 further supported the relevance of DNE- and valence-related variables for burnout. Removing all DNE and valence variables from the model significantly worsened model fit (*χ*²_10_=28.5; *P*=.002). Similarly, excluding all DNE variables (*χ²*_9_=28.0; *P*=.001) or valence alone (*χ²*_4_=13.6; *P*=.009) significantly reduced model fit, whereas excluding daytime DNE variables alone did not significantly worsen model fit (*χ*²_3_=6.2; *P*=.10).

A broadly similar pattern was observed for engagement. Removing all DNE and valence variables from the model significantly worsened model fit (*χ*²_10_=23.7; *P*=.009). Similarly, excluding all DNE variables (*χ²*_9_=23.5; *P*=.005), daytime DNE variables alone (*χ*²_3_=9.1; *P*=.03), or valence alone (*χ*²_4_=10.3; *P*=.04) significantly reduced model fit.

**Table 3.** Model comparison results for burnout and engagement. Model comparisons were conducted using likelihood ratio tests based on maximum likelihood estimation. Lower-order models were compared against the full model.

Model comparison	LRT^a^ chi-square (*df*), *P* value
Burnout model comparison	
Full model vs model without EDA^b^ and valence	28.5 (10), .002
Full model vs model without daytime EDA	6.2 (3), .10
Full model vs model without EDA	28.0 (9), .001
Full model vs model without valence	13.6 (4), .009
Engagement model comparison	
Full model vs model without EDA and valence	23.7 (10), .009
Full model vs model without daytime EDA	9.1 (3), .03
Full model vs model without EDA	23.5 (9), .005
Full model vs model without valence	10.3 (4), .04

a LRT: likelihood ratio test.

b EDA: electrodermal activity.

### Illustrative Model-Based Profiles

Illustrative model-based profiles for hypothetical combinations of arousal and valence are presented in Table 4 and Figure 5. Across both burnout and engagement outcomes, valence shifted predicted values in the expected direction, with negative valence associated with higher predicted burnout scores and lower predicted engagement scores compared with positive valence.

**Table 4.** Illustrative model-based profiles across arousal and valence conditions^a^.

Condition	BAT-12^b^ model-based estimate (80% PI^c^)	UWES-9^d^ model-based estimate (80% PI)
High arousal + negative valence	3.84 (2.34 to 5.34)	0.01 (−3.27 to 3.30)
High arousal + positive valence	2.04 (1.18 to 2.89)	4.37 (2.48 to 6.25)
Low arousal + negative valence	3.50 (2.74 to 4.25)	1.82 (0.16 to 3.48)
Low arousal + positive valence	1.88 (1.26 to 2.51)	4.80 (3.41 to 6.20)

a Model-based estimates of BAT-12 and UWES-9 total scores with asymptotic 80% prediction intervals. Because the Gaussian linear model is unbounded, prediction intervals may extend beyond the theoretical ranges of the BAT-12 and UWES-9 scales. The intervals are used descriptively to illustrate uncertainty around the hypothetical model-based profiles and should not be interpreted as feasible questionnaire scores or as evidence of predictive accuracy.

b BAT-12: 12-item Burnout Assessment Tool.

c PI: prediction interval.

d UWES-9: 9-item Utrecht Work Engagement Scale.

**Figure 5.**
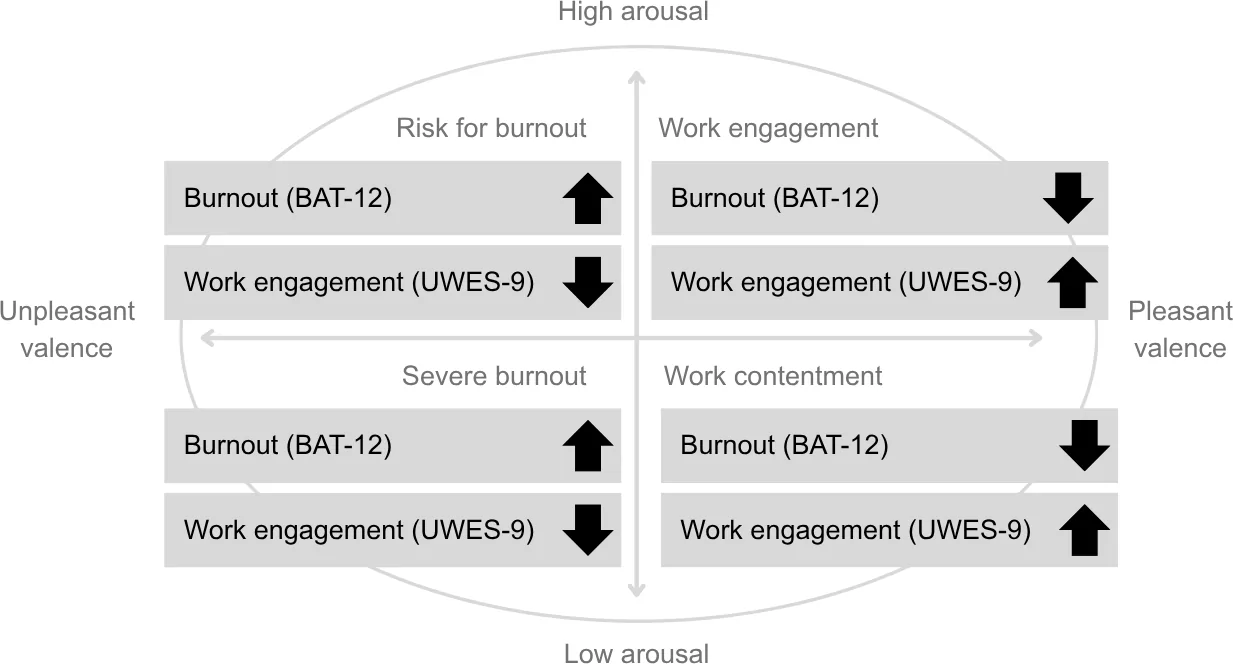
Illustrative model-based profiles across hypothetical combinations of arousal and valence. The profiles visualize fitted model relationships and should not be interpreted as evidence of predictive accuracy, individual-level prediction, or clinically actionable classification. Exact model-based estimates and corresponding 80% prediction intervals are shown in Table 4. BAT-12: 12-item Burnout Assessment Tool; UWES-9: 9-item Utrecht Work Engagement Scale.

Under high arousal conditions, predicted burnout scores were higher when valence was negative (BAT-12=3.84) than when it was positive (BAT-12=2.04). A similar pattern was observed under low arousal conditions, although the differences were smaller (BAT-12=3.50 vs 1.88). Correspondingly, predicted engagement scores were lowest under combinations of negative valence and high arousal (UWES-9=0.01) and highest under positive valence conditions (UWES-9=4.37‐4.80). Prediction intervals were wide and partially overlapping, indicating uncertainty in the magnitude of the estimated effects. These profiles illustrate the fitted model relationships and were not intended to assess predictive accuracy or individual-level classification.

### Time-Resolved Analyses

Time-resolved analyses showed that hourly DNE profiles differed across burnout and engagement subgroups (Figures 6A and 6B; Tables S4 and S5 in Multimedia Appendix 1). For burnout, the at-risk subgroup showed higher DNE levels than the no-risk subgroup mainly from midday to midnight. The at-risk subgroup also showed higher DNE levels than the likely burnout subgroup from late morning to late evening. In contrast, differences between the no-risk and likely burnout subgroups were not statistically significant in the hour-level comparisons. For engagement, subgroup differences varied across the 24-hour cycle. The low or very low engagement subgroup tended to show higher daytime DNE levels than the moderate or high engagement subgroups, although statistically significant differences were limited to selected hours. The very high engagement subgroup also differed from the moderate and high engagement subgroups during late-evening and early-night hours. No statistically significant differences were observed between the low or very low and very high engagement subgroups in the hour-level comparisons.

**Figure 6.**
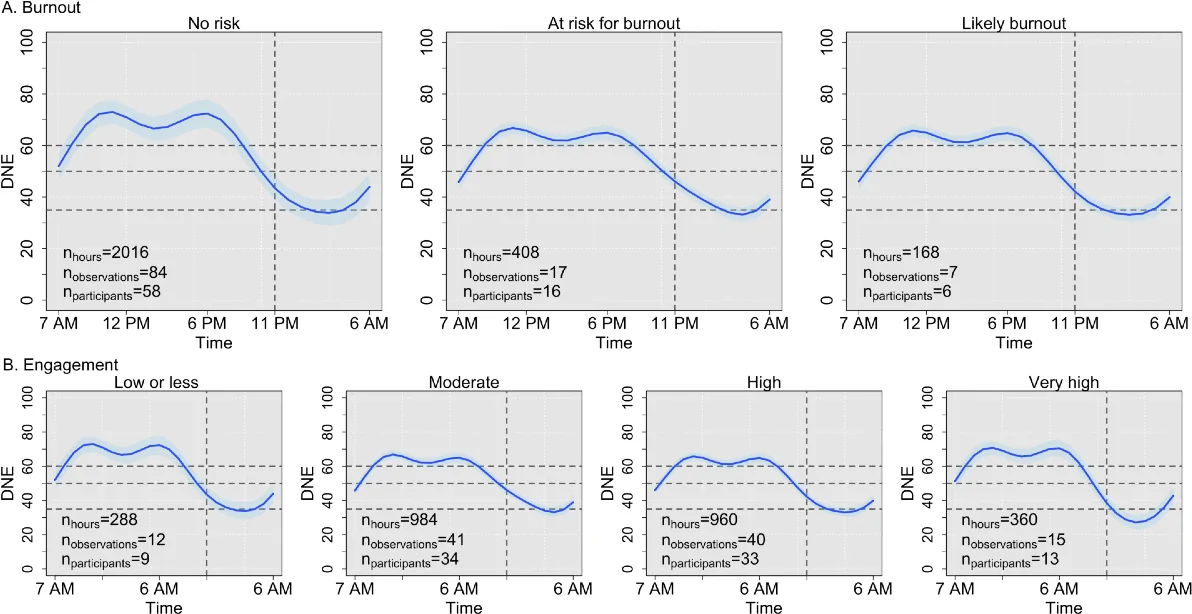
Expected hourly marginal means over a 24-hour period within burnout and engagement subgroups. (A) burnout and (B) engagement. Estimated marginal means and pointwise 95% CIs for double normalized electrodermal activity by hour of day from a mixed-effects model accounting for within-subject correlation. Subgroups of observations based on burnout and engagement total score classification. Anxiety and depression were controlled to their minimal level. The dashed horizontal lines=daily around 50, daytime around 60, and nighttime around 35. The dashed vertical line indicates the transition from daytime (7 AM to 11 PM) to nighttime (11 PM to 7 AM).

## Discussion

### Principal Findings

This study examined the use of continuous wearable EDA together with mEMA in a naturalistic occupational setting and evaluated associations between the proprietary DNE index, valence, burnout, and engagement. The protocol yielded analyzable multimodal data from repeated 14-day monitoring periods, providing practical evidence that this type of data collection can be implemented in real-world settings.

EDA-derived indices were associated with burnout and engagement but do not provide construct-specific measurement of these states. Although several DNE-related associations were only near-significant, these associations emerged despite adjustment for a large number of potentially correlated covariates, including depressive symptoms. This suggests that DNE-related variables may capture aspects of burnout not fully explained by conventional psychological or behavioral measures. However, because the model included multiple correlated DNE variables and interaction terms, the observed interaction patterns should be interpreted cautiously. Including these variables improved model fit relative to models without them, indicating that ambulatory physiological signals and momentary self-reports provide complementary information. In both outcomes, the association between EDA-derived arousal and burnout or engagement depended on valence, suggesting that arousal alone is insufficient to distinguish between these states.

Time-resolved analyses suggested that occupational well-being was not reflected merely in static average levels of autonomic activation but also in the timing and shape of daily DNE profiles. Burnout-related differences were most evident from midday to late evening, with the at-risk group showing the highest activation. Engagement-related differences appeared nonlinear: moderate and high engagement were associated with lower daytime DNE than both low or very low and very high engagement. This suggests that engagement may not follow a simple “more engagement–less arousal” gradient but may instead be linked to a more balanced daily activation profile. Overall, the results support the feasibility of integrating wearable EDA sensing with mEMA to capture real-world patterns of occupational well-being and highlight the importance of considering both arousal and valence in interpreting these patterns.

### Interpretation of Findings

The present findings extend prior psychophysiological research by demonstrating that continuous, real-world monitoring of autonomic activation can provide meaningful insights into occupational well-being. The observed diurnal variation in the DNE index—characterized by higher daytime and lower nighttime levels—was consistent with previously described temporal variation in EDA [41-43]. This pattern provides physiological plausibility for the DNE signal but does not by itself establish construct-specific validity or full biomarker validation, as behavioral activity, sleep timing, environmental conditions, wear behavior, and features of the proprietary normalization procedure may also contribute to the observed variation.

The association between EDA-derived arousal and both burnout and engagement aligns with the circumplex model of occupational well-being, which conceptualizes these states along dimensions of arousal and valence [21-23]. Importantly, the findings indicate that both burnout and engagement may involve elevated levels of arousal, differing primarily in their associated valence. This supports a view that high-arousal states in occupational contexts are not inherently positive or negative but must be interpreted in relation to subjective experience. The moderating role of valence in the regression models further underscores the importance of combining physiological and self-reported data to differentiate between qualitatively distinct states that may share similar levels of autonomic activation.

The time-of-day–specific patterns observed in subgroup analyses provide additional insight into the dynamic nature of occupational well-being. Elevated evening and early-night arousal among individuals at higher risk of burnout may reflect prolonged activation and insufficient recovery, consistent with theories of allostatic load [44] and the effort-recovery model [45]. Similarly, the nonmonotonic relationship between engagement and arousal suggests that both very low and very high engagement may be associated with elevated activation, whereas moderate levels of engagement may reflect a more balanced physiological state. These findings are in line with previous research indicating that engagement, while generally considered beneficial, may also involve increased physiological demands under certain conditions [16].

Contrary to our initial expectation, higher levels of burnout were not associated with reduced autonomic activation. Instead, individuals in the likely burnout subgroup showed elevated DNE levels, particularly during the late-evening and early-night hours. Although findings regarding physiological correlates of burnout remain inconsistent, several studies have reported autonomic dysregulation characterized by elevated sympathetic activity and reduced parasympathetic functioning among individuals with burnout [46,47]. In contrast, a systematic review by de Looff et al [18] concluded that while higher job stress was consistently associated with increased sympathetic activity, evidence regarding burnout remained inconclusive because of methodological heterogeneity and inconsistent findings across studies. Our findings add to this literature by suggesting that elevated autonomic activation may also characterize individuals at higher risk of burnout in naturalistic occupational settings.

An alternative explanation relates to differences in measurement methodology. As noted by de Looff et al [18], psychophysiological burnout studies vary considerably in recording duration, measurement context, analytical procedures, and physiological outcome measures, making comparisons difficult. Most previous studies relied on resting assessments, laboratory stress protocols, or monitoring periods lasting only hours or a few days. Continuous ambulatory monitoring across 14 consecutive days may capture temporal patterns of autonomic activation and recovery that shorter assessments cannot detect. Notably, the most pronounced differences between burnout subgroups in this study emerged during late-evening and early-night hours, suggesting that alterations in recovery-related autonomic regulation may be particularly relevant to burnout risk.

The absence of reduced activation is also noteworthy because our original expectation was partly informed by literature linking burnout and depression, including evidence of overlapping symptoms and shared underlying mechanisms [48,49]. Reduced physiological reactivity and blunted stress responses have been reported in depression and in some clinical burnout populations, particularly among individuals with severe exhaustion or comorbid affective disorders [50-52]. However, the present sample consisted of actively working knowledge workers rather than patients with clinically diagnosed burnout, and symptoms of depression and anxiety were statistically controlled in the regression analyses. It is therefore possible that the physiological profile observed here reflects an earlier stage of the burnout process characterized by sustained sympathetic activation rather than physiological hypoactivation. Future studies including clinically burned-out populations are needed to determine whether autonomic activation changes across different stages of burnout development.

Taken together, these findings suggest that continuous physiological monitoring captures patterns of autonomic activation that are meaningfully associated with occupational well-being but are not specific to individual psychological constructs. Instead, EDA appears to reflect general arousal dynamics, which, when combined with contextual or self-reported information such as valence, can provide a more nuanced understanding of burnout and engagement in real-world settings.

### Implications for Digital Health and Occupational Well-Being Monitoring

The present findings highlight the potential of integrating wearable physiological sensing and mEMA into digital health approaches for monitoring occupational well-being in real-world settings. Continuous EDA provided a low-burden means of capturing patterns of autonomic activation over extended periods, while EMA-based assessments of valence offered complementary contextual information. Together, these modalities enabled a more nuanced characterization of burnout and engagement than either approach alone. Our findings align with recent work suggesting that wearable and EMA-based monitoring may capture meaningful longitudinal variation in occupational well-being in naturalistic settings [3].

An important methodological aspect of this study relates to the use of a proprietary, device-derived EDA index. Rather than evaluating a fully transparent physiological signal-processing pipeline, this study examines the behavior of a commercially implemented EDA-derived arousal metric under naturalistic conditions. This distinction reflects a broader consideration in digital health research, where many scalable and user-facing technologies rely on proprietary algorithms. In this context, this study provides preliminary convergent evidence from the observed behavior of the DNE index and its associations with established self-report measures of occupational well-being. The observed diurnal pattern provides physiological plausibility but does not by itself establish construct-specific validity or full biomarker validation. More broadly, the findings illustrate both the potential and the limitations of evaluating proprietary sensing systems when the underlying processing pipeline is not fully accessible.

At the same time, this approach entails important trade-offs. Limited computational transparency constrains direct comparability with studies using open EDA processing methods and highlights the need for continued validation of wearable-derived indices across populations and contexts. Future research should seek to bridge this gap by combining proprietary systems with complementary validation approaches, including multimodal sensing and open physiological benchmarks.

From a digital health perspective, such approaches may enable more timely and proactive monitoring of occupational well-being compared with traditional metrics, such as sick leave or employee turnover, which typically reflect deterioration after it has already occurred. Continuous physiological and self-reported data may help identify patterns of sustained activation and insufficient recovery that are not readily captured by periodic self-report surveys.

However, the use of wearable and mobile technologies in occupational contexts raises important ethical considerations. Physiological data are inherently sensitive, and their use in workplace settings may introduce risks related to privacy, perceived surveillance, and potential misuse of data for performance evaluation or discrimination. To mitigate these risks, the implementation of such systems should be guided by principles of transparency, voluntary participation, and clear separation between health monitoring and organizational decision-making processes.

In particular, future applications could include individual feedback and self-management approaches. However, the present findings are based on group-level associations and do not establish the reliability or diagnostic usage of these measures at the individual level. Future research with larger samples is needed before such applications can be validated. Aggregated and anonymized data may also be used to inform organizational practices, provided that appropriate safeguards are in place.

The findings also suggest that high levels of engagement may be associated with elevated physiological activation. Although engagement is generally considered beneficial, sustained high-arousal states may also require adequate recovery. These findings highlight the importance of balancing performance and recovery in digital health interventions. Rather than focusing solely on maximizing engagement, future systems should support sustainable patterns of work by integrating physiological signals with contextual and subjective information.

Overall, wearable and mobile technologies offer promising opportunities for workplace contexts, but their responsible use requires careful consideration of ethical, legal, and social implications to ensure that benefits to individuals and organizations are achieved without compromising individual privacy, autonomy, and trust.

### Strengths and Limitations

This study has several strengths. First, it used continuous, long-term physiological monitoring in real-world settings, enabling the assessment of autonomic activation patterns over extended periods rather than relying on laboratory-based or short-term measurements. Second, the combination of wearable EDA and mEMA allowed for the integration of physiological and subjective data, providing a more comprehensive view of occupational well-being. Third, the longitudinal design, including 2 measurement waves, enhanced the robustness of the findings and allowed examination of repeated observations within individuals.

Several limitations should also be considered. The final analytic sample was smaller than initially anticipated due to incomplete data across the study components, including insufficient EDA wear time, missing mEMA responses, and noncompletion of the postmeasurement questionnaire. Given the number of fixed-effect terms and the inclusion of related DNE summaries and interaction terms, individual coefficient-level findings should be interpreted cautiously and together with their confidence intervals and the model comparison results. Because burnout and engagement were assessed after the monitoring period, these measures were unavailable for participants who did not complete the questionnaire, and it was not possible to determine whether attrition was systematically related to the outcomes of interest. However, the observed distribution of burnout levels in the sample was broadly consistent with reported prevalence estimates in the Finnish working population, suggesting that substantial bias due to selective attrition is unlikely, although it cannot be ruled out.

The self-report measures of burnout and engagement were designed to capture experiences over the same 2-week period as the physiological monitoring and were completed shortly after the measurement period. However, some participants completed the questionnaires with a delay (median 3 days, mean 5 days, maximum 17 days), introducing a potential temporal mismatch between physiological data and self-reported outcomes. Given that burnout and engagement are typically conceptualized as relatively stable constructs rather than day-to-day states, this delay is unlikely to have materially affected the observed associations, although some attenuation of relationships cannot be ruled out.

The administration of mEMA every 3 hours was reported as burdensome by some participants, which may have reduced compliance and limited variability in valence responses. In addition, the 3-hour retrospective reporting window may have attenuated short-lived fluctuations in affect. Future studies could explore less burdensome mEMA designs, such as reduced prompt frequency or simplified response formats, to improve adherence and sensitivity to momentary changes. The study relied on assumptions regarding typical daily schedules, including fixed working hours and sleep periods, which may not accurately reflect individual variations, particularly in occupations involving flexible or irregular work patterns. This may have introduced some imprecision in distinguishing between rest and activity periods.

Importantly, the DNE index is derived from a proprietary algorithm and cannot be independently reconstructed from raw EDA signals. The archived research data contain DNE outputs from the minute level through the measurement period summary variables, together with the R code used for study-specific data processing and statistical analyses. Thus, the derivation of the study-specific DNE summaries and the statistical analyses can be reproduced from the archived DNE output, whereas the proprietary transformation from sensor-level EDA to DNE cannot be independently reproduced. Exact replication of the original data acquisition pipeline is further limited because the specific device and software version used in this study is no longer supported. However, the device and DNE-processing configuration remained unchanged during the study, reducing the possibility that differences between measurement waves were attributable to device or software updates.

The proprietary nature of the DNE algorithm also highlights the importance of longitudinal validation across device generations. Future studies should evaluate whether newer hardware and software versions produce comparable DNE profiles and maintain similar associations with occupational well-being outcomes. Future research should aim to replicate these findings in larger and more diverse samples, including clinical populations with severe burnout. Longitudinal studies with finer temporal alignment between physiological and self-report measures would help clarify the temporal dynamics of autonomic activation in relation to occupational well-being. Furthermore, continued development and validation of wearable-derived indices, along with integration of multimodal data sources, may enhance the interpretability and applicability of digital health approaches for monitoring mental well-being in real-world contexts.

### Conclusions

This study provides exploratory evidence that continuous wearable EDA monitoring can be combined with mEMA in naturalistic occupational settings. DNE-derived measures of autonomic arousal were associated with burnout and engagement, while mEMA-based valence provided complementary information for interpreting these associations.

The findings support further investigation of proprietary EDA-derived arousal metrics as candidate digital biomarkers but do not by themselves establish DNE as a fully validated, construct-specific biomarker of burnout or engagement. The study was not designed to establish prospective predictive performance, screening or diagnostic validity, sensitivity to change, or individual-level decision usage.

More broadly, the findings illustrate both the potential and the limitations of evaluating proprietary wearable-derived measures in real-world digital health research. Further studies using independent physiological reference measures, larger and more diverse populations, and prospective validation designs are needed before clinical or occupational applications can be considered.

## Supplementary material

10.2196/104167 Multimedia Appendix 1 Supplementary tables on psychological measures, participant characteristics, data availability, pairwise comparisons, and model-based prediction profiles.

## Abbreviations

BAT-12: 12-item Burnout Assessment Tool
BDI-II: Beck Depression Inventory-II
DNE: double normalized electrodermal activity
EDA: electrodermal activity
EMA: ecological momentary assessment
GLS: generalized least squares
mEMA: mobile-based ecological momentary assessment
REML: restricted maximum likelihood
UWES-9: 9-item Utrecht Work Engagement Scale
GAD-7: Generalized Anxiety Disorder 7-item scale
