# Supplementary material for: Evaluating Wearable Electrodermal Activity as a Candidate Digital Biomarker of Occupational Well-Being: Longitudinal Real-World Study
Source: JMIR Form Res. 2026 Nov 11;10:e104167. doi: 10.2196/104167 (PMC13754084; doi:10.2196/104167)
Supplement: Supplementary file 1 [file formative-v10-e104167-s001.docx]

## Multimedia Appendix 1

Table S1. Scales and cut-off values used to control for anxiety and depression.

| **Scale** | **Score** | **Cut-Off Values** |
| --- | --- | --- |
| Generalized Anxiety Disorder (GAD-7) | Minimal | ≤4 |
|  | Mild | >4 and ≤9 |
|  | Moderate | >9 and ≤14 |
|  | Severe | >14 |
| Beck Depression Inventory (BDI-II) | Minimal | ≤13 |
|  | Mild | >13 and ≤19 |
|  | Moderate | >19 and ≤28 |
|  | Severe | >28 |

Anxiety and depression were assessed to screen for clinically relevant symptoms and to establish the healthy control group. Anxiety was assessed using the 7-item Generalized Anxiety Disorder scale (GAD-7), with items rated on a 4-point scale from 0 (not at all) to 3 (nearly every day), yielding a total score of 0–21. Scores were classified as minimal (≤4), mild (5–9), moderate (10–14), or severe (≥15) [1]. Depression was assessed using the 21-item Beck Depression Inventory-II (BDI-II). Scores were classified as minimal (≤13), mild (14–19), moderate (20–28), or severe (≥29) [2].

Table S2. Control variables and their average values.

| **Variable** | **Mean (SD)_1_** | **Mean (SD)_2_** |
| --- | --- | --- |
| Physical activity. Physical activity rate was estimated by  dividing step count measured with the smart ring by total  minutes of wearing the smart ring. | 3.6 (1.2) | 3.5 (1.2) |
| Age in years at the start of the study. | 39.2 (7.0) | 39.7 (7.3) |
| Gender |  |  |
| Female | 32 | 26 |
| Male | 25 | 25 |
| Working hours per week. | 37.5 (4.7) | 36.8 (4.5) |
| Family commitments to underaged people household  members. |  |  |
| Yes | 31 | 24 |
| No | 26 | 27 |
| Family commitments to adult household members or older  adult relatives |  |  |
| Yes | 2 | 1 |
| No | 55 | 50 |
| Occurrence of major life events in the past six months. |  |  |
| Yes | 8 | 12 |
| No | 49 | 39 |
| Experienced need for a positive change in one’s work and  lifestyle in the past six months. |  |  |
| Yes | 45 | 39 |
| No | 12 | 12 |
| Received support from occupational health care/supervisor/colleague(s)/other social relations/themself/Nuanic measurement for better stress management. |  |  |
| Totally or Partly agree (Yes) | 51 | 47 |
| Totally or Partly disagree, Indifferent or No need (No) | 6 | 4 |
| Self-reported average hours of sleep per day during the week | 7.2 (1.0) | 7.2 (1.0) |
| Self-reported average hours of sleep per day at weekends | 8.0 (1.0) | 8.0 (1.1) |
| Use of medication affecting SNS activity |  |  |
| Reducing | 0 | 0 |
| No (known) effect | 53 | 48 |
| Activates | 4 | 3 |
| Managerial position |  |  |
| Yes | 9 | 6 |
| No | 48 | 45 |
| Experience of working in current position in years | 7.1 (6.8) | 6.1 (5.7) |
| Use of nicotine products daily or at least daily |  |  |
| Yes | 3 | 2 |
| No | 54 | 49 |
| Body Mass Index | 26.2 (5.7) | 25.7 (3.6) |
| GAD-7 total score | 5.4 (4.1) | 5.8 (3.7) |
| BDI-II total score | 8.7 (6.7) | 9.4 (7.5) |
| Number of observations | 57 | 51 |

*Note*. _1_ = Wave 1 (3/2022); _2_ = Wave 2 (9/2022)

SNS: sympathetic nervous system

Table S3. Data availability by study component and measurement wave.

| **Study component** | **Wave 1 (3/2022)** | **Wave 2 (9/2022)** | **Total observations** |
| --- | --- | --- | --- |
| Wearable EDA | 68 | 62 | 130 |
| mEMA valence | 96 | 74 | 170 |
| Post-measurement survey | 93 | 65 | 158 |
| Complete datasets | 57 | 51 | 108 |
| *Note*. Valid EDA data were available for 68 participants in wave 1 and 62 participants in wave 2. Valence data were available for 96 and 74 participants, respectively, and post-measurement survey data for 93 and 65 participants, respectively. Complete datasets requiring valid observations from all three sources within the same wave were available for 57 participant-wave observations in wave 1 and 51 participant-wave observations in wave 2 (108 observations in total). These observations were obtained from 75 distinct participants, of whom 33 contributed data in both waves, 24 only in wave 1, and 18 only in wave 2. | | | |

Table S4. Pairwise comparisons of burnout (BAT-12) group marginal means.

| **Contrast** | **Hour** | **Estimate** | **SE** | **df** | **t.ratio** | ***P*** |
| --- | --- | --- | --- | --- | --- | --- |
| No risk - (At-risk) | 0 | -6,51 | 2,08 | 2491 | -3,14 | .005 |
| No risk - Likely burnout | 0 | -1,13 | 4,23 | 2491 | -0,27 | .96 |
| (At-risk) - Likely burnout | 0 | 5,38 | 4,35 | 2491 | 1.24 | .43 |
| No risk - (At-risk) | 1 | -4.72 | 2.10 | 2491 | -2.24 | .06 |
| No risk - Likely burnout | 1 | -3.78 | 4.26 | 2491 | -0.89 | .65 |
| (At-risk) - Likely burnout | 1 | 0.94 | 4.38 | 2491 | 0.22 | .98 |
| No risk - (At-risk) | 2 | -2.30 | 2.18 | 2491 | -1.06 | .54 |
| No risk - Likely burnout | 2 | -5.66 | 4.34 | 2491 | -1.30 | .39 |
| (At-risk) - Likely burnout | 2 | -3.36 | 4.47 | 2491 | -0.75 | .73 |
| No risk - (At-risk) | 3 | 0.12 | 2.28 | 2491 | 0.05 | >.99 |
| No risk - Likely burnout | 3 | -6.42 | 4.46 | 2491 | -1.44 | .32 |
| (At-risk) - Likely burnout | 3 | -6.54 | 4.58 | 2491 | -1.43 | .33 |
| No risk - (At-risk) | 4 | 1.85 | 2.38 | 2491 | 0.78 | .73 |
| No risk - Likely burnout | 4 | -5.95 | 4.57 | 2491 | -1.30 | .39 |
| (At-risk) - Likely burnout | 4 | -7.80 | 4.70 | 2491 | -1.66 | .22 |
| No risk - (At-risk) | 5 | 2.38 | 2.45 | 2491 | 0.97 | .60 |
| No risk - Likely burnout | 5 | -4.46 | 4.65 | 2491 | -0.96 | .60 |
| (At-risk) - Likely burnout | 5 | -6.84 | 4.78 | 2491 | -1.43 | .33 |
| No risk - (At-risk) | 6 | 1.67 | 2.48 | 2491 | 0.67 | .78 |
| No risk - Likely burnout | 6 | -2.31 | 4.67 | 2491 | -0.49 | .87 |
| (At-risk) - Likely burnout | 6 | -3.98 | 4.81 | 2491 | -0.83 | 0.69 |
| No risk - (At-risk) | 7 | 0.11 | 2.45 | 2491 | 0.04 | >.99 |
| No risk - Likely burnout | 7 | 0.05 | 4.65 | 2491 | 0.01 | >.99 |
| (At-risk) - Likely burnout | 7 | -0.06 | 4.78 | 2491 | -0.01 | >.99 |
| No risk - (At-risk) | 8 | -1.69 | 2.38 | 2491 | -0.71 | .76 |
| No risk - Likely burnout | 8 | 2.26 | 4.57 | 2491 | 0.49 | .87 |
| (At-risk) - Likely burnout | 8 | 3.95 | 4.70 | 2491 | 0.84 | .68 |
| No risk - (At-risk) | 9 | -3.18 | 2.28 | 2491 | -1.39 | .35 |
| No risk - Likely burnout | 9 | 4.06 | 4.46 | 2491 | 0.91 | .63 |
| (At-risk) - Likely burnout | 9 | 7.25 | 4.58 | 2491 | 1.58 | .25 |
| No risk - (At-risk) | 10 | -4.13 | 2.18 | 2491 | -1.89 | .14 |
| No risk - Likely burnout | 10 | 5.37 | 4.34 | 2491 | 1.24 | .43 |
| (At-risk) - Likely burnout | 10 | 9.49 | 4.47 | 2491 | 2.13 | .09 |
| No risk - (At-risk) | 11 | -4.64 | 2.10 | 2491 | -2.21 | .07 |
| No risk - Likely burnout | 11 | 6.17 | 4.26 | 2491 | 1.45 | .32 |
| (At-risk) - Likely burnout | 11 | 10.81 | 4.38 | 2491 | 2.47 | .04 |
| No risk - (At-risk) | 12 | -5.05 | 2.08 | 2491 | -2.43 | .04 |
| No risk - Likely burnout | 12 | 6.54 | 4.23 | 2491 | 1.55 | .27 |
| (At-risk) - Likely burnout | 12 | 11.59 | 4.35 | 2491 | 2.66 | .02 |
| No risk - (At-risk) | 13 | -5.63 | 2.10 | 2491 | -2.67 | .02 |
| No risk - Likely burnout | 13 | 6.59 | 4.26 | 2491 | 1.55 | .27 |
| (At-risk) - Likely burnout | 13 | 12.22 | 4.38 | 2491 | 2.79 | .02 |
| No risk - (At-risk) | 14 | -6.44 | 2.18 | 2491 | -2.95 | .009 |
| No risk - Likely burnout | 14 | 6.49 | 4.34 | 2491 | 1.49 | .29 |
| (At-risk) - Likely burnout | 14 | 12.93 | 4.47 | 2491 | 2.89 | .01 |
| No risk - (At-risk) | 15 | -7.29 | 2.28 | 2491 | -3.19 | .004 |
| No risk - Likely burnout | 15 | 6.40 | 4.46 | 2491 | 1.44 | .32 |
| (At-risk) - Likely burnout | 15 | 13.69 | 4.58 | 2491 | 2.99 | .008 |
| No risk - (At-risk) | 16 | -7.90 | 2.38 | 2491 | -3.32 | .003 |
| No risk - Likely burnout | 16 | 6.45 | 4.57 | 2491 | 1.41 | .34 |
| (At-risk) - Likely burnout | 16 | 14.35 | 4.70 | 2491 | 3.06 | .006 |
| No risk - (At-risk) | 17 | -8.07 | 2.45 | 2491 | -3.29 | .003 |
| No risk - Likely burnout | 17 | 6.70 | 4.65 | 2491 | 1.44 | .32 |
| (At-risk) - Likely burnout | 17 | 14.77 | 4.78 | 2491 | 3.09 | .006 |
| No risk - (At-risk) | 18 | -7.84 | 2.48 | 2491 | -3.17 | .004 |
| No risk - Likely burnout | 18 | 7.06 | 4.67 | 2491 | 1.51 | .27 |
| (At-risk) - Likely burnout | 18 | 14.90 | 4.81 | 2491 | 3.10 | .006 |
| No risk - (At-risk) | 19 | -7.49 | 2.45 | 2491 | -3.06 | .006 |
| No risk - Likely burnout | 19 | 7.29 | 4.65 | 2491 | 1.57 | .26 |
| (At-risk) - Likely burnout | 19 | 14.78 | 4.78 | 2491 | 3.09 | .006 |
| No risk - (At-risk) | 20 | -7.30 | 2.38 | 2491 | -3.06 | .006 |
| No risk - Likely burnout | 20 | 7.07 | 4.57 | 2491 | 1.55 | .27 |
| (At-risk) - Likely burnout | 20 | 14.37 | 4.70 | 2491 | 3.06 | .006 |
| No risk - (At-risk) | 21 | -7.38 | 2.28 | 2491 | -3.23 | .004 |
| No risk - Likely burnout | 21 | 6.11 | 4.46 | 2491 | 1.37 | .36 |
| (At-risk) - Likely burnout | 21 | 13.49 | 4.58 | 2491 | 2.94 | .009 |
| No risk - (At-risk) | 22 | -7.55 | 2.18 | 2491 | -3.46 | .002 |
| No risk - Likely burnout | 22 | 4.29 | 4.34 | 2491 | 0.99 | .59 |
| (At-risk) - Likely burnout | 22 | 11.84 | 4.47 | 2491 | 2.65 | .02 |
| No risk - (At-risk) | 23 | -7.40 | 2.10 | 2491 | -3.52 | .001 |
| No risk - Likely burnout | 23 | 1.74 | 4.26 | 2491 | 0.41 | .91 |
| (At-risk) - Likely burnout | 23 | 9.15 | 4.38 | 2491 | 2.09 | .09 |

Table S5. Pairwise comparisons of UWES-9 group marginal means.

| **Contrast** | | **Hour** | | **Estimate** | | **SE** | | **df** | | **t.ratio** | | ***P*** | |
| --- | --- | --- | --- | --- | --- | --- | --- | --- | --- | --- | --- | --- | --- |
| Low or less - Moderate | 0 | | -3.46 | | 2.36 | | 2482 | | -1.47 | | .46 | |  |
| Low or less - High | 0 | | 0.89 | | 2.44 | | 2482 | | 0.37 | | .98 | |  |
| Low or less - Very high | 0 | | 6.42 | | 2.96 | | 2482 | | 2.17 | | .13 | |  |
| Moderate - High | 0 | | 4.35 | | 1.40 | | 2482 | | 3.11 | | .01 | |  |
| Moderate - Very high | 0 | | 9.88 | | 2.24 | | 2482 | | 4.41 | | <.001 | |  |
| High - Very high | 0 | | 5.52 | | 2.19 | | 2482 | | 2.52 | | .06 | |  |
| Low or less - Moderate | 1 | | -3.16 | | 2.39 | | 2482 | | -1.32 | | .55 | |  |
| Low or less - High | 1 | | 0.70 | | 2.49 | | 2482 | | 0.28 | | >.99 | |  |
| Low or less - Very high | 1 | | 7.23 | | 3.02 | | 2482 | | 2.40 | | .08 | |  |
| Moderate - High | 1 | | 3.86 | | 1.42 | | 2482 | | 2.72 | | .03 | |  |
| Moderate - Very high | 1 | | 10.39 | | 2.28 | | 2482 | | 4.56 | | <.001 | |  |
| High - Very high | 1 | | 6.53 | | 2.22 | | 2482 | | 2.94 | | .02 | |  |
| Low or less - Moderate | 2 | | -1.95 | | 2.48 | | 2482 | | -0.78 | | .86 | |  |
| Low or less - High | 2 | | 0.61 | | 2.60 | | 2482 | | 0.23 | | >.99 | |  |
| Low or less - Very high | 2 | | 7.10 | | 3.15 | | 2482 | | 2.25 | | .11 | |  |
| Moderate - High | 2 | | 2.56 | | 1.47 | | 2482 | | 1.73 | | .31 | |  |
| Moderate - Very high | 2 | | 9.04 | | 2.38 | | 2482 | | 3.79 | | <.001 | |  |
| High - Very high | 2 | | 6.49 | | 2.31 | | 2482 | | 2.81 | | .03 | |  |
| Low or less - Moderate | 3 | | -0.22 | | 2.60 | | 2482 | | -0.08 | | >.99 | |  |
| Low or less - High | 3 | | 0.70 | | 2.74 | | 2482 | | 0.25 | | >.99 | |  |
| Low or less - Very high | 3 | | 5.96 | | 3.34 | | 2482 | | 1.79 | | .28 | |  |
| Moderate - High | 3 | | 0.92 | | 1.55 | | 2482 | | 0.59 | | .93 | |  |
| Moderate - Very high | 3 | | 6.18 | | 2.52 | | 2482 | | 2.45 | | .07 | |  |
| High - Very high | 3 | | 5.26 | | 2.42 | | 2482 | | 2.18 | | .13 | |  |
| Low or less - Moderate | 4 | | 1.65 | | 2.72 | | 2482 | | 0.61 | | .93 | |  |
| Low or less - High | 4 | | 1.20 | | 2.88 | | 2482 | | 0.42 | | .98 | |  |
| Low or less - Very high | 4 | | 4.19 | | 3.51 | | 2482 | | 1.19 | | .63 | |  |
| Moderate - High | 4 | | -0.45 | | 1.61 | | 2482 | | -0.28 | | >.99 | |  |
| Moderate - Very high | 4 | | 2.53 | | 2.65 | | 2482 | | 0.96 | | .77 | |  |
| High - Very high | 4 | | 2.99 | | 2.53 | | 2482 | | 1.18 | | .64 | |  |
| Low or less - Moderate | 5 | | 3.42 | | 2.80 | | 2482 | | 1.22 | | .61 | |  |
| Low or less - High | 5 | | 2.32 | | 2.98 | | 2482 | | 0.78 | | .87 | |  |
| Low or less - Very high | 5 | | 2.38 | | 3.63 | | 2482 | | 0.66 | | .91 | |  |
| Moderate - High | 5 | | -1.10 | | 1.66 | | 2482 | | -0.66 | | .91 | |  |
| Moderate - Very high | 5 | | -1.04 | | 2.74 | | 2482 | | -0.38 | | .98 | |  |
| High - Very high | 5 | | 0.06 | | 2.60 | | 2482 | | 0.02 | | >.99 | |  |
| Low or less - Moderate | 6 | | 4.95 | | 2.83 | | 2482 | | 1.75 | | .30 | |  |
| Low or less - High | 6 | | 4.02 | | 3.02 | | 2482 | | 1.33 | | .54 | |  |
| Low or less - Very high | 6 | | 1.12 | | 3.67 | | 2482 | | 0.31 | | >.99 | |  |
| Moderate - High | 6 | | -0.93 | | 1.68 | | 2482 | | -0.56 | | .95 | |  |
| Moderate - Very high | 6 | | -3.83 | | 2.77 | | 2482 | | -1.38 | | .51 | |  |
| High - Very high | 6 | | -2.89 | | 2.63 | | 2482 | | -1.10 | | .69 | |  |
| Low or less - Moderate | 7 | | 6.16 | | 2.80 | | 2482 | | 2.20 | | .12 | |  |
| Low or less - High | 7 | | 5.96 | | 2.98 | | 2482 | | 2.00 | | .19 | |  |
| Low or less - Very high | 7 | | 0.72 | | 3.63 | | 2482 | | 0.20 | | >.99 | |  |
| Moderate - High | 7 | | -0.20 | | 1.66 | | 2482 | | -0.12 | | >.99 | |  |
| Moderate - Very high | 7 | | -5.44 | | 2.74 | | 2482 | | -1.99 | | .19 | |  |
| High - Very high | 7 | | -5.24 | | 2.60 | | 2482 | | -2.01 | | .18 | |  |
| Low or less - Moderate | 8 | | 6.95 | | 2.72 | | 2482 | | 2.56 | | .05 | |  |
| Low or less - High | 8 | | 7.59 | | 2.88 | | 2482 | | 2.63 | | .04 | |  |
| Low or less - Very high | 8 | | 1.06 | | 3.51 | | 2482 | | 0.30 | | >.99 | |  |
| Moderate - High | 8 | | 0.64 | | 1.61 | | 2482 | | 0.40 | | .98 | |  |
| Moderate - Very high | 8 | | -5.89 | | 2.65 | | 2482 | | -2.22 | | .18 | |  |
| High - Very high | 8 | | -6.54 | | 2.53 | | 2482 | | -2.59 | | .048 | |  |
| Low or less - Moderate | 9 | | 7.21 | | 2.60 | | 2482 | | 2.77 | | .03 | |  |
| Low or less - High | 9 | | 8.41 | | 2.74 | | 2482 | | 3.06 | | .01 | |  |
| Low or less - Very high | 9 | | 1.72 | | 3.34 | | 2482 | | 0.51 | | .96 | |  |
| Moderate - High | 9 | | 1.20 | | 1.55 | | 2482 | | 0.77 | | .87 | |  |
| Moderate - Very high | 9 | | -5.49 | | 2.52 | | 2482 | | -2.18 | | .17 | |  |
| High - Very high | 9 | | -6.69 | | 2.42 | | 2482 | | -2.77 | | .03 | |  |
| Low or less - Moderate | 10 | | 6.90 | | 2.48 | | 2482 | | 2.78 | | .03 | |  |
| Low or less - High | 10 | | 8.20 | | 2.60 | | 2482 | | 3.15 | | .009 | |  |
| Low or less - Very high | 10 | | 2.21 | | 3.15 | | 2482 | | 0.70 | | .90 | |  |
| Moderate - High | 10 | | 1.30 | | 1.47 | | 2482 | | 0.88 | | .81 | |  |
| Moderate - Very high | 10 | | -4.68 | | 2.38 | | 2482 | | -1.96 | | .20 | |  |
| High - Very high | 10 | | -5.99 | | 2.31 | | 2482 | | -2.59 | | .047 | |  |
| Low or less - Moderate | 11 | | 6.13 | | 2.39 | | 2482 | | 2.56 | | .051 | |  |
| Low or less - High | 11 | | 7.20 | | 2.49 | | 2482 | | 2.90 | | .02 | |  |
| Low or less - Very high | 11 | | 2.25 | | 3.02 | | 2482 | | 0.75 | | .88 | |  |
| Moderate - High | 11 | | 1.07 | | 1.42 | | 2482 | | 0.75 | | .88 | |  |
| Moderate - Very high | 11 | | -3.88 | | 2.28 | | 2482 | | -1.70 | | .32 | |  |
| High - Very high | 11 | | -4.95 | | 2.22 | | 2482 | | -2.23 | | .12 | |  |
| Low or less - Moderate | 12 | | 5.22 | | 2.36 | | 2482 | | 2.21 | | .12 | |  |
| Low or less - High | 12 | | 5.98 | | 2.44 | | 2482 | | 2.45 | | .07 | |  |
| Low or less - Very high | 12 | | 1.85 | | 2.96 | | 2482 | | 0.62 | | .92 | |  |
| Moderate - High | 12 | | 0.76 | | 1.40 | | 2482 | | 0.54 | | .95 | |  |
| Moderate - Very high | 12 | | -3.37 | | 2.24 | | 2482 | | -1.51 | | .43 | |  |
| High - Very high | 12 | | -4.13 | | 2.19 | | 2482 | | -1.89 | | .23 | |  |
| Low or less - Moderate | 13 | | 4.57 | | 2.39 | | 2482 | | 1.91 | | .22 | |  |
| Low or less - High | 13 | | 5.17 | | 2.49 | | 2482 | | 2.08 | | .16 | |  |
| Low or less - Very high | 13 | | 1.28 | | 3.02 | | 2482 | | 0.43 | | .97 | |  |
| Moderate - High | 13 | | 0.60 | | 1.42 | | 2482 | | 0.42 | | .96 | |  |
| Moderate - Very high | 13 | | -3.29 | | 2.28 | | 2482 | | -1.44 | | .47 | |  |
| High - Very high | 13 | | -3.89 | | 2.22 | | 2482 | | -1.75 | | .30 | |  |
| Low or less - Moderate | 14 | | 4.54 | | 2.48 | | 2482 | | 1.83 | | .26 | |  |
| Low or less - High | 14 | | 5.16 | | 2.60 | | 2482 | | 1.99 | | .19 | |  |
| Low or less - Very high | 14 | | 0.90 | | 3.15 | | 2482 | | 0.28 | | >.99 | |  |
| Moderate - High | 14 | | 0.62 | | 1.47 | | 2482 | | 0.42 | | .97 | |  |
| Moderate - Very high | 14 | | -3.64 | | 2.38 | | 2482 | | -1.53 | | .42 | |  |
| High - Very high | 14 | | -4.26 | | 2.31 | | 2482 | | -1.85 | | .25 | |  |
| Low or less - Moderate | 15 | | 5.19 | | 2.60 | | 2482 | | 1.99 | | .19 | |  |
| Low or less - High | 15 | | 5.89 | | 2.74 | | 2482 | | 2.15 | | .14 | |  |
| Low or less - Very high | 15 | | 0.88 | | 3.34 | | 2482 | | 0.26 | | >.99 | |  |
| Moderate - High | 15 | | 0.71 | | 1.55 | | 2482 | | 0.46 | | .97 | |  |
| Moderate - Very high | 15 | | -4.31 | | 2.52 | | 2482 | | -1.71 | | .32 | |  |
| High - Very high | 15 | | -5.01 | | 2.42 | | 2482 | | -2.07 | | .16 | |  |
| Low or less - Moderate | 16 | | 6.26 | | 2.72 | | 2482 | | 2.30 | | .10 | |  |
| Low or less - High | 16 | | 6.92 | | 2.88 | | 2482 | | 2.40 | | .08 | |  |
| Low or less - Very high | 16 | | 1.18 | | 3.51 | | 2482 | | 0.34 | | .99 | |  |
| Moderate - High | 16 | | 0.66 | | 1.61 | | 2482 | | 0.41 | | .98 | |  |
| Moderate - Very high | 16 | | -5.07 | | 2.65 | | 2482 | | -1.91 | | .22 | |  |
| High - Very high | 16 | | -5.73 | | 2.53 | | 2482 | | -2.27 | | .11 | |  |
| Low or less - Moderate | 17 | | 7.21 | | 2.80 | | 2482 | | 2.58 | | .049 | |  |
| Low or less - High | 17 | | 7.62 | | 2.98 | | 2482 | | 2.56 | | .052 | |  |
| Low or less - Very high | 17 | | 1.60 | | 3.63 | | 2482 | | 0.44 | | .97 | |  |
| Moderate - High | 17 | | 0.41 | | 1.66 | | 2482 | | 0.25 | | >.99 | |  |
| Moderate - Very high | 17 | | -5.62 | | 2.74 | | 2482 | | -2.05 | | .17 | |  |
| High - Very high | 17 | | -6.03 | | 2.60 | | 2482 | | -2.32 | | .10 | |  |
| Low or less - Moderate | 18 | | 7.45 | | 2.83 | | 2482 | | 2.64 | | .04 | |  |
| Low or less - High | 18 | | 7.55 | | 3.02 | | 2482 | | 2.50 | | .06 | |  |
| Low or less - Very high | 18 | | 1.90 | | 3.67 | | 2482 | | 0.52 | | .96 | |  |
| Moderate - High | 18 | | 0.10 | | 1.68 | | 2482 | | 0.06 | | >.99 | |  |
| Moderate - Very high | 18 | | -5.55 | | 2.77 | | 2482 | | -2.00 | | .19 | |  |
| High - Very high | 18 | | -5.65 | | 2.63 | | 2482 | | -2.15 | | .14 | |  |
| Low or less - Moderate | 19 | | 6.59 | | 2.80 | | 2482 | | 2.35 | | .09 | |  |
| Low or less - High | 19 | | 6.61 | | 2.98 | | 2482 | | 2.22 | | .12 | |  |
| Low or less - Very high | 19 | | 2.06 | | 3.63 | | 2482 | | 0.57 | | .94 | |  |
| Moderate - High | 19 | | 0.02 | | 1.66 | | 2482 | | 0.01 | | >.99 | |  |
| Moderate - Very high | 19 | | -4.53 | | 2.74 | | 2482 | | -1.65 | | .35 | |  |
| High - Very high | 19 | | -4.55 | | 2.60 | | 2482 | | -1.75 | | .30 | |  |
| Low or less - Moderate | 20 | | 4.62 | | 2.72 | | 2482 | | 1.70 | | .32 | |  |
| Low or less - High | 20 | | 5.08 | | 2.88 | | 2482 | | 1.76 | | .29 | |  |
| Low or less - Very high | 20 | | 2.24 | | 3.51 | | 2482 | | 0.64 | | .92 | |  |
| Moderate - High | 20 | | 0.45 | | 1.61 | | 2482 | | 0.28 | | >.99 | |  |
| Moderate - Very high | 20 | | -2.38 | | 2.65 | | 2482 | | -0.90 | | .81 | |  |
| High - Very high | 20 | | -2.83 | | 2.53 | | 2482 | | -1.12 | | .68 | |  |
| Low or less - Moderate | 21 | | 1.99 | | 2.60 | | 2482 | | 0.76 | | .87 | |  |
| Low or less - High | 21 | | 3.44 | | 2.74 | | 2482 | | 1.25 | | .59 | |  |
| Low or less - Very high | 21 | | 2.73 | | 3.34 | | 2482 | | 0.82 | | .85 | |  |
| Moderate - High | 21 | | 1.45 | | 1.55 | | 2482 | | 0.94 | | .78 | |  |
| Moderate - Very high | 21 | | 0.75 | | 2.52 | | 2482 | | 0.30 | | >.99 | |  |
| High - Very high | 21 | | -0.71 | | 2.42 | | 2482 | | -0.29 | | >.99 | |  |
| Low or less - Moderate | 22 | | -0.64 | | 2.48 | | 2482 | | -0.26 | | >.99 | |  |
| Low or less - High | 22 | | 2.12 | | 2.60 | | 2482 | | 0.82 | | .85 | |  |
| Low or less - Very high | 22 | | 3.71 | | 3.15 | | 2482 | | 1.18 | | .64 | |  |
| Moderate - High | 22 | | 2.77 | | 1.47 | | 2482 | | 1.88 | | .24 | |  |
| Moderate - Very high | 22 | | 4.35 | | 2.38 | | 2482 | | 1.83 | | .26 | |  |
| High - Very high | 22 | | 1.58 | | 2.31 | | 2482 | | 0.69 | | .90 | |  |
| Low or less - Moderate | 23 | | -2.60 | | 2.39 | | 2482 | | -1.09 | | .70 | |  |
| Low or less - High | 23 | | 1.30 | | 2.49 | | 2482 | | 0.52 | | .95 | |  |
| Low or less - Very high | 23 | | 5.07 | | 3.02 | | 2482 | | 1.68 | | .33 | |  |
| Moderate - High | 23 | | 3.90 | | 1.42 | | 2482 | | 2.75 | | .03 | |  |
| Moderate - Very high | 23 | | 7.67 | | 2.28 | | 2482 | | 3.36 | | .004 | |  |
| High - Very high | 23 | | 3.77 | | 2.22 | | 2482 | | 1.69 | | .33 | |  |

Table S6. Predictor values used for model-based predictions and prediction intervals.

| Explanatory variable | BAT-12 | | UWES-9 | |
| --- | --- | --- | --- | --- |
|  | High arousal | Low arousal | High arousal | Low arousal |
| Daily DNE level | 58 | 43 | 58 | 43 |
| Daily DNE level variation | 6.5 | 6.5 | 6.5 | 6.5 |
| Nighttime DNE level | 48 | 38 | 48 | 38 |
| Within nighttime DNE variation | 35 | 35 | 35 | 35 |
| Daytime DNE level | 70 | 48 | 70 | 48 |
| Within daytime DNE variation | 16 | 16 | 16 | 16 |
| Physical activity | 3.6 | 3.6 | 3.6 | 3.6 |
| Age | 39.2 | 39.2 | 39.2 | 39.2 |
| Sex | Female | Female | Female | Female |
| Working hours per week | 37.5 | 37.5 | 37.5 | 37.5 |
| Children | Yes | Yes | Yes | Yes |
| Responsibilities adults | No | No | No | No |
| Major life event | No | No | No | No |
| Need change | Yes | Yes | Yes | Yes |
| Intervention | Yes | Yes | Yes | Yes |
| Sleep time work | 7.2 | 7.2 | 7.2 | 7.2 |
| Sleep time leisure | 8 | 8 | 8 | 8 |
| Medication | No | No | No | No |
| Supervisor | No | No | No | No |
| Experience, years | 7.1 | 7.1 | 7.1 | 7.1 |
| Nicotine | No | No | No | No |
| Body Mass Index | 26.2 | 26.2 | 26.2 | 26.2 |
| GAD-7 total score | 5.4 | 5.4 | 5.4 | 5.4 |
| BDI-II total score | 8.7 | 8.7 | 8.7 | 8.7 |

References

1.	Spitzer RL, Kroenke K, Williams JBW, Löwe B. A Brief Measure for Assessing Generalized Anxiety Disorder: The GAD-7. Arch Intern Med 2006 May 22;166(10):1092–1097. PMID:16717171

2.	Beck AT, Steer RA, Brown GK. BDI-II, Beck Depression Inventory Manual. 2nd ed. San Antonio, TX: Psychological Corporation; 1996.
